# Characterization and Evolution of the Cell Cycle-Associated Mob Domain-Containing Proteins in Eukaryotes

**Published:** 2007-08-08

**Authors:** Nicola Vitulo, Alessandro Vezzi, Giulio Galla, Sandra Citterio, Giada Marino, Benedetto Ruperti, Monica Zermiani, Emidio Albertini, Giorgio Valle, Gianni Barcaccia

**Affiliations:** 1 Dipartimento di Biologia, University of Padova, Viale G. Colombo 3, 35121, Padova.; 2 Dipartimento di Agronomia Ambientale e Produzioni Vegetali, University of Padova - Agripolis, Viale dell’Università 16, 35020, Legnaro, Padova, Italy.; 3 Dipartimento di Scienze dell’Ambiente e del Territorio, University of Milano - Bicocca, Piazza della Scienza 1, 20126, Milano, Italy.; 4 Dipartimento di Scienze Agrarie e Ambientali, University of Udine, Via delle Scienze 208, 33100, Udine, Italy.; 5 Dipartimento di Biologia Vegetale e Biotecnologie Agroambientali e Zootecniche, Borgo XX Giugno, 06121, Perugia, Italy.

**Keywords:** Mob genes, protein structure, phylogenesis, cytokinesis, apoptosis, morphogenesis

## Abstract

The MOB family includes a group of cell cycle-associated proteins highly conserved throughout eukaryotes, whose founding members are implicated in mitotic exit and co-ordination of cell cycle progression with cell polarity and morphogenesis. Here we report the characterization and evolution of the MOB domain-containing proteins as inferred from the 43 eukaryotic genomes so far sequenced. We show that genes for Mob-like proteins are present in at least 41 of these genomes, confirming the universal distribution of this protein family and suggesting its prominent biological function. The phylogenetic analysis reveals five distinct MOB domain classes, showing a progressive expansion of this family from unicellular to multicellular organisms, reaching the highest number in mammals. Plant Mob genes appear to have evolved from a single ancestor, most likely after the loss of one or more genes during the early stage of Viridiplantae evolutionary history. Three of the Mob classes are widespread among most of the analyzed organisms. The possible biological and molecular function of Mob proteins and their role in conserved signaling pathways related to cell proliferation, cell death and cell polarity are also presented and critically discussed.

## Introduction

Normal development of multicellular organisms requires appropriate cell numbers and organ sizes, and it is determined by coordinated cell proliferation, cell growth and programmed cell death (reviewed by Danial and Korsmeyer, 2004; Murray, 2004; Sherr, 2004). Disruption or malfunction of these processes can cause diseases, such as cancer. Recent studies in yeasts and higher eukaryotes have led to the identification of a number of proteins and their interactors as key components of specific metabolic pathways that control the coordination between cell proliferation, morphogenesis and programmed cell death (Lai et al. 2005).

Members of the NDR (nuclear Dbf2-related) family, a subclass of AGC-type protein kinases, are essential components of pathways that control important cellular processes, such as mitotic exit, cytokinesis, cell proliferation and morphogenesis, and apoptosis (reviewed by Hergovich et al. 2006). Some recent progress in this field has shed light on the mechanisms that underlie the regulation and function of the NDR proteins by means of the co-activator Mob (Mps1-one binder) proteins. Combined data from yeast, worms, flies, mice and human cells have highlighted the conserved and important roles of MOB-domain containing proteins in the activation of NDR kinases (Manning et al. 2002; Hergovich et al. 2006). In particular, Mob proteins play a critical role in cell-cycle regulation chiefly by interacting with and activating the Dbf2-related protein kinases (Komarnitsky et al. 1998; Lee et al. 2001; Mah et al. 2001). This subfamily of serine/threonine kinases includes Dbf2, Dbf20 and Cbk1 in *Saccharomyces cerevisiae*, Ndr1, Ndr2, Lats1 and Lats2 in human, Warts (aka dLats) and Trc (aka dNdr) in *Drosophila melanogaster* and Sax1 (aka ceNdr) and a hypothetical Lats homolog in *Caenorhabditis elegans*. Like their Mob protein partners, this subfamily of protein kinases regulates cell growth, cell division and cell morphology (Justice et al. 1995; Xu et al. 1995; Zallen et al. 2000). In metazoans, members of the NDR family act as tumour suppressors (for example, LATS1) or potential protooncogenes (for example, NDR1). In the molecular regulation of the NDR family kinases, an important role is also played by protein kinases belonging to the sterile 20 (STE20)-like kinase group (for review see Hergovich et al. 2006). A summary of available information on Mob-domain containing proteins and its interacting NDR-type kinases in given in Table 1.

Mob proteins interact with NDR kinases by binding a conserved stretch of primary sequence at their N terminus, also known as NTR (N-terminal regulatory) domain. The interaction of Mob proteins with the NTR activation site is a conserved feature of all members of the NDR-kinase family that have been tested so far in yeasts, flies and human cells (Mrkobrada et al. 2006). Interestingly, Mob proteins do not function solely as co-activators of NDR kinases, but are also required for the localization of yeast NDR kinases. Recent evidence further indicates that the targeting of Mob proteins to the plasma membrane is sufficient to fully activate mammalian NDR1/2 (Hergovich et al. 2005; Stegert et al. 2005) and LATS1 (Hergovich et al. 2006). Taken together, these findings indicate that Mob binding to the N terminus of NDR family members allows efficient auto-phosphorylation on the activation segment, and at the same time recruits NDRs to activation sites, thereby bringing this protein into close proximity with its upstream activating kinase.

The MOB family includes a group of cell cycle-associated, non-catalytic proteins highly conserved in eukaryotes, whose founding members are implicated in mitotic exit and co-ordination of cell polarity with cell cycle progression (Luca et al. 2001; Stegmeier et al. 2002). Two distinct Mob proteins, Mob1 and Mob2, are known in fungi, while an expansion in metazoans gives rise to six in human, four in *D. melanogaster*, and four in *C. elegans* (Mrkobrada et al. 2006). Mob1 proteins have been demonstrated to be important for both mitosis completion and cell plate formation in yeast (Luca and Winey, 1998; Salimova et al. 2000). Moreover, the Mob1-related proteins Mob2 physically associates with specific kinases throughout the cell cycle, being required and periodically activated in yeast to promote polarized growth (Weiss et al. 2002; Nelson et al. 2003). Mob1-like proteins have been also found in animals (Stavridi et al. 2003; Ponchon et al. 2004; Devroe et al. 2004). Plant genomes such as alfalfa, rice and *Arabidopsis* contain uncharacterized Mob1-related genes (Van Damme et al. 2004; Citterio et al. 2005, 2006). Although there are data to suggest that Mob proteins act as kinase activating subunits in higher eukaryotes, their function remains to be proved.

This paper deals with the characterization and evolution of the cell cycle-associated and morphogenesis-related MOB domain-containing proteins belonging to 43 eukaryotic genomes. Results on the structural characteristics and phylogenesis of Mob proteins are reported, and adopted for the classification of family members using a novel nomenclature. The biological and molecular function of Mob proteins and their role in conserved signaling pathways related to cell proliferation, cell death and cell polarity are also presented and critically discussed.

## Methods for Bioinformatic Analyses

To perform a complete and exhaustive analysis on the Mob domain distribution and phylogenetic relationship among eukaria, the proteomes of 43 complete and ongoing eukaryotic genomes were downloaded from NCBI (ftp://ftp.ncbi.nih.gov/genomes/), ENSEMBL (ftp://ftp.ensembl.org/pub) and DOE Joint Genome Institute (http://genome.jgi-psf.org/euk_home.html) sites.

The hidden Markov model profile for the Mob domain (Pfam code: PF03637) was downloaded from the Pfam site (http://www.sanger.ac.uk/Software/Pfam/) (Sonnhammer et al. 1998) and was used to search for similarity against the proteome databases using HMMER software (Durbin et al. 1998).

Using a cut-off expectation value equal or lower than e^−20^, a total of 202 MOB domain containing proteins were identified (see supplementary Table 1S). Among these, ten sequences were not considered in the subsequent analysis because of low quality problems. As many as 192 Mob domains were extracted from the original sequences and aligned using the progressive alignment algorithm implemented in CLUSTALW (Higgins et al. 1992), and the result was edited to remove any ambiguous region.

The ProtTest software (http://darwin.uvigo.es/) (Abascal et al. 2005) was used to select the most appropriate amino acid substitution models for tree construction. Phylogenetic tree was generated from Mob domain amino acid sequences using the linux version of PhyML (Guindon et al. 2003) with JTT+I+G as protein model evolution and with a bootstrap analysis of 200 re-sampling runs.

The phylogenetic analysis allowed the identification of different Mob groups. The proteins belonging to different branches of the phylogenetic tree were aligned using CLUSTALW software and a consensus sequence was extracted for each group. The consensus sequences reflect the most common sequences in the alignment. For a more detailed analysis and visualization of each aligned group, a web logo was created using the web version of WebLogo software (http://weblogo.berkeley.edu).

## Results: Structural Analysis of Mob Proteins

### Primary structure characteristics and classification of family members

Mob proteins are a small family of highly conserved proteins, found in all eukaryotes, approximately 210 to 240 amino acid residues in length. The evolution of MOB family genes is poorly understood and a classification and nomenclature of Mob genes is not fully established. Here we propose some insight into the evolutionary dynamics of this family and a system of classification based on a phylogenetic analysis of Mob genes in all complete and ongoing eukaryotic genome sequences.

Mrkobrada et al. (2006) proposed a classification based on the alignment of the core domain of Mob proteins from yeast to human, identifying three distinct groups defined by similarity between the conserved N-terminal region. On the basis of the distribution of ScMob1 and ScMob2 members within the clusters, they referred to the groups as Mob1-like, Mob2-like and Mob3-like. The Mob1-like group contains two subgroups (A and B): Mob1A contains the ortholog of ScMob1 in fungal species and single proteins from *H. sapiens* and *D. melanogaster*, whereas the Mob1B group contains one or more Mob proteins from *H. sapiens*, *D. melanogaster*, *D. rerio, C. elegans* and *X. laevis*. The Mob2-like cluster contains two groups, Mob2A, consisting of the fungal ortholog ScMob2 and a second group, Mob2B, containing metazoan genes. Finally, the Mob3-like group is the most divergent one and contains a single protein from each metazoan organism analyzed. Moreover, two mammalian homologs to yeast MOB genes have been described, the mammalian Mob homolog (MMh), that has high similarity with *S. cerevisiae* Mob2 genes, and phocein or mammalian Mob1 distantly related to MOB1 and MOB2 (Hennebold et al. 2000; Baillat et al. 2001; 2002; Moreno et al. 2001). Stavridi et al. (2003) proposed that MMh be referred to as Mob2 and that phocein/mMob1 be referred to only as phocein.

**Figure 1.**
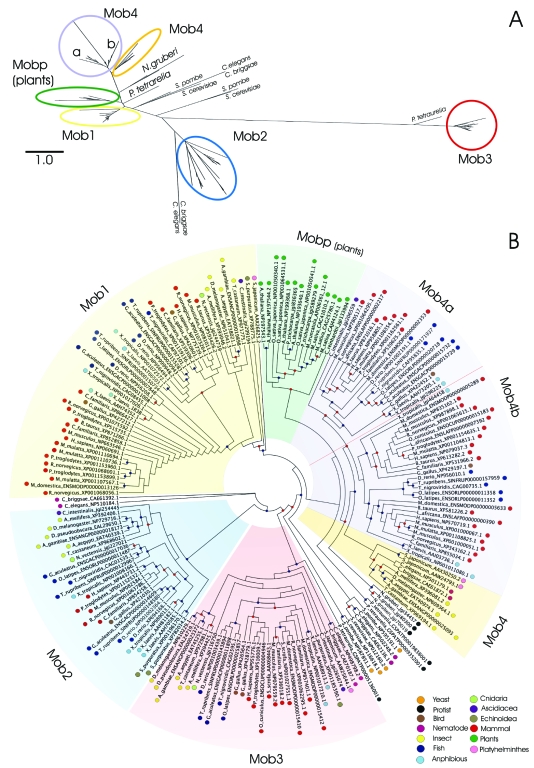
Phylogenetic tree of the 192 Mob domain proteins. Mob groups identified with the phylogenetic analysis are shown and highlighted in different colors. The Panel **A** shows a maximum likelihood Mob protein phylogenetic tree (the scale represents the number of amino acid substitution per site). The Panel **B** shows a maximum likelihood cladogram without branch length for an easier visualization of the Mob groups (the colored dot on each organism name refers to the taxonomy classification). The red dot on each node of the tree represents a bootstrap value equal or higher than 50%, while the blue dot a bootstrap value equal or higher than 70%.

To classify the Mob domain into related groups of sequences, a phylogenetic analysis was performed, by searching Mob domain hidden Markov model profile on all complete or ongoing available eukaryotic genomes. Figure 1 shows the phylogenetic tree for 192 Mob genes (see also supplementary Figure 1S). The results highlight that Mob domain is clearly separated into five classes: Mob1, Mob2, Mob3, Mob4 and Mobp with high bootstrap support. Among the different classes, Mob3 is the most divergent clade.

The numbers of genes in class Mob1, Mob2, Mob3, Mob4 and Mobp are 47, 28, 31, 57 and 14 respectively. Some of the *C. elegans* and *C. briggsae*, and *S. cerevisiae*, *S. pombe* and Protist Mob related proteins clustered outside these groups and they will be treated separately. Mob4 class can be subdivided into two phylogenetic clades, corresponding to invertebrate (9 genes) and vertebrate Mob-like genes (48). Moreover, vertebrate Mob-like genes can be further subdivided into other two subgroups, Mob4a, containing 19 genes, and Mob4b with 29 Mob-like proteins.

The average amino acid identity within Mob classes is 92% (Mob1), 54% (Mob2), 86% (Mob3), 70% (Mob4), 86% (Mob4a), 84% (Mob4b) and 78% (Mobp).

The results partially support the previous classification by Mrkobrada et al. (2006). The main differences are probably due to the higher number of genes analyzed in this study and concern the Mob1 class which was previously subdivided into two groups, Mob1A and Mob1B. Our analysis allowed us to recognize a Mob1 class that corresponds to Mob1A group and a Mob4 class that contains the previously established Mob1B group (see Mrkobrada et al. 2006). Moreover, both Mob4a and Mob4b groups proved to contain Mob-like genes previously annotated as part of the Mob1B group (Mrkobrada et al. 2006).

## Phylogenesis: Distribution and Evolution of Mob Genes in Eukaryotic Genomes

The phylogenetic tree shown in Figure 1 has been generated from the available proteomes of 43 complete and draft genomes (see also supplementary Figure 1S). Only in two plant genomes, *Ostreococcus tauri* and *Zea mays*, it was not possible to identify Mob-like proteins. This could be due to the consensus sequence quality and to the genome assembly; both of them being quite important issues for producing a high quality alignment and a reliable counting of Mob genes.

**Figure 2.**
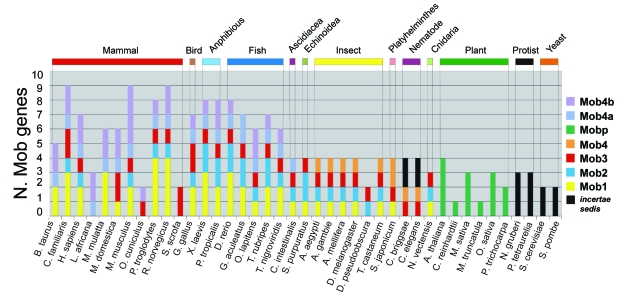
Mob protein distribution among organisms used in the analysis. Different Mob groups are represented with different color and the species grouped on the base of the taxonomy classification. The label “*incertae sedis*” refers to Mob proteins that have an undefined position on the phylogenetic tree.

Figure 2 shows the distribution of Mob-like proteins among the organisms used for the analysis. Vertebrates (mammals, birds, amphibian and fish) have the highest number of Mob genes, distributed in all the Mob classes. Interestingly, all the vertebrate genes of the Mob4 class are included in a single branch that is supported by a bootstrap value of 77%. This suggests that all Mob4-like vertebrate genes derived from a single ancestral gene at the basis of Mob4 chordata/hemichordata gene evolution. The two subclasses Mob4a and Mob4b found in vertebrates must have arisen from an early duplication, which further subdivided this class into two subgroups.

Among vertebrates, mammals reveal the highest number of Mob genes. *M. musculus* have the highest number of Mob4b genes (4), while *P. troglodytes* and *R. norvegicus* have the highest number of Mob1 genes (4). *L. africana*, *O. cuniculus* and *S. scrofa*, compared to the other mammals, present a smaller number of Mob genes, probably reflecting a still limited coverage of the entire gene space of these organisms.

Mrkobrada et al. (2006) reports that the *Homo sapiens* genome contains six Mob-like proteins whereas in our analysis we found seven Mob-like proteins. Nomenclature of Mob genes not only is poorly established but often can be quite misleading. Proteins identified by codes NP_060691 and NP_775739 are annotated as “Mob4B” and “MOB1, Mps One Binder kinase activator-like 1A” respectively, while in our phylogenetic tree they both fall in Mob1 group. NP_443731 is a member of the Mob2 group but it is annotated as “HCCA2 protein”. Moreover protein NP_955776 in public databases is defined as “preimplantation protein 3 isoform 2” and in our analysis belongs to the Mob3 group. Finally, NP_958805, NP_079037, NP_570719 proteins, annotated respectively as “MOB1, Mps One Binder kinase activator-like 2C isoform 2”, “MOB1, Mps One Binder kinase activator-like 2B” and “MOB-LAK”, are all members of the Mob4 group, with the first one belonging to Mob4a and the last two to Mob4b group.

All insects show four Mob genes belonging respectively to Mob1, Mob2, Mob3 and Mob4 classes, except *D. pseudoobscura*, in which only two Mob genes can be found, probably due to genome assembly quality. Finally, plants represent a monophyletic group defined as Mobp class.

The phylogenetic tree shows that *S. cereviseae* (NP_012160, NP_116618), *S. pombe* (NP_595191, NP_587851), *C. elegans* (NP_502248, NP_510184), *C. briggsae* (CAE62136, CAE61392) and Protist proteins are listed as *incertae sedis*. Because of historical reasons, in the previous literature Mob yeast genes have been generally described as the founding members of the Mob family (Stavridi et al. 2003, Mrkobrada et al. 2006). However, the protein sequences analyzed in this work, mostly of multicellular organisms, do not allow a clear definition of the phylogenetic relationships existing among the yeast and the other Mob genes. In this regard it is interesting to point out that NP_116618 and NP_587851 yeast proteins, described as Mob2A in Mrkobrada et al. (2006), did not cluster with any other protein, possibly due to an early divergence of these orthologs in the lineage that generated modern Fungi.

Even if it is quite difficult to reconstruct the evolution of the Mob family as a whole, some possible scenarios can be drawn by looking at the distribution of genes in the so far sequenced organisms. If plants are not considered, Figure 2 indicates a minimum of two genes in all the eukaryotic genomes analyzed. This in turn seems to suggest a duplication of the ancestral Mob gene at an early stage of the eukaryotic evolution.

Going from unicellular to multicellular organisms there is a progressive expansion of the Mob family, reaching the highest number in mammals. Moreover, plant Mob-like genes appear to have evolved from a single ancestor, most likely due to the loss of one or more genes during the early evolution of Viridiplantae. Compared to vertebrates, plants show a significant decrease in Mob-like gene possibly due to the adaptation to a much more simple life style. The relationship observed among genes of the same organism and/or different organisms suggests that the Mob gene family evolved under a birth-and-death type of evolution. In this model new genes are created by duplication, and some duplicated genes are maintained in the genome for a long time whereas other are deleted or become nonfunctional through deleterious mutations (Nei and Rooney, 2005).

## Mob-like Protein Structure and Architecture of Mob-domain Containing Proteins

Three Mob1 protein structures have been described in literature. Human and *Xenopus laevis* structures correspond to the most conserved C-terminal core but lack the variable N-terminal region, whereas *Saccharomyces* Mob1 structure contains both the conserved C-terminal core and the variable N-terminal region (Stavridi et al. 2003; Ponchon et al. 2004; Mrkobrada et al. 2006).

In our phylogenetic tree, Human and *Xenopus* proteins used in structure analyses belong to the Mob1 group, while *Saccharomyces* Mob-like proteins have been assigned as *incertae sedis*.

**Figure 3.**
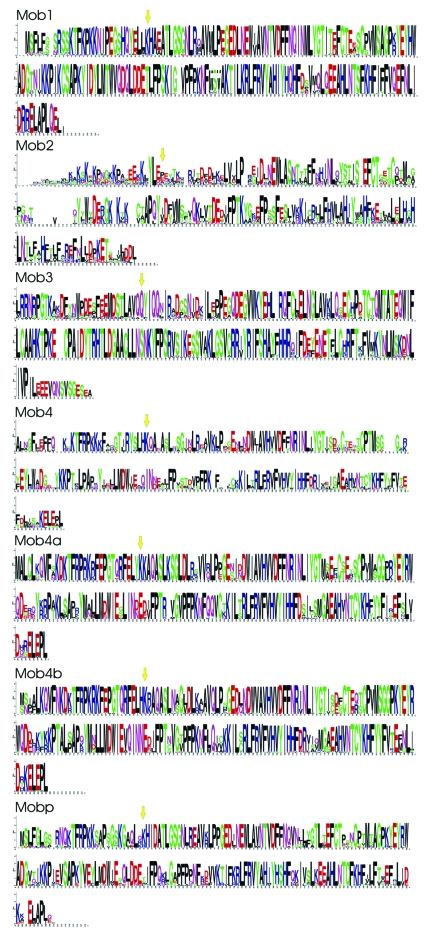
Sequence logos for each of the multiple alignment Mob groups: Mob1, Mob2, Mob3, Mob4, Mob4a, Mob4b and Mobp. Each logo consists of stacks of symbols, one stack for each position in the sequence: the overall height of the stack indicates the sequence conservation at that position, while the height of symbols within the stack indicates the relative frequency of each amino acid at that position. The yellow arrows represent the starting position adopted for the multiple alignment of Mob group consensus sequences.

**Figure 4.**
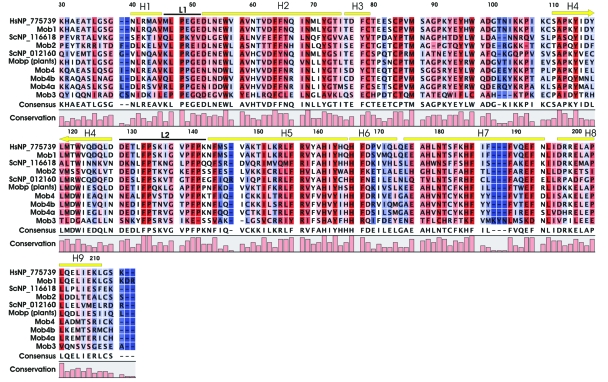
Multiple alignment of Mob group consensus sequences. The alignment was performed taking into consideration two structural defined Mob proteins, Hs NP_775739 and Sc NP_012160 plus Sc NP_116618. The helix (yellow lines) and loops (black lines) nomenclature and position on the alignment refer to Hs Mob protein as described by *Stavridi* et al. (2005). On each of the alignment columns, a colour scale going from red to blue represents high and low amino acid conservation, respectively.

To compare the different Mob classes, a consensus sequence for each identified group was constructed. Figure 3 shows the amino acid sequence conservation over all positions for each of the seven Mob groups: Mob1, Mob2, Mob3, Mob4, Mob4a, Mob4b and Mobp. These consensus sequences were then adopted to generate a new multiple protein alignment, using three additional Mob proteins, such as the *S. cerevisiae* Mob1 and Mob2 proteins (NP_116618 and NP_012160) and one *H. sapiens* Mob1 protein (NP_775739). The latter two proteins were added in the alignment since they have been structurally characterized (Stavridi et al. 2003; Mrkobrada et al. 2006). The final multiple alignment of Mob group consensus sequences in shown in Figure 4.

Mob proteins are approximately 210 to 240 amino acid residues in length, with the exception of *S. cerevisiae* Mob1, which has a further 78 residue N-terminal extension not conserved or even present in the closely related fungal proteins.

Mob1 adopts a globular structure consisting of seven α helices, two 3_10_-helices and a β hairpin. The core of the structure consists of a helical bundle formed by four long α helices (H2, H4, H5, and H7). This left-handed four-helix bundle, comprising the H2 and H5 helices running anti-parallel to H4 and H7 helices, is capped at one end by two short helices (H3 and H6) and the β hairpin, which are stabilized to the helical bundle via a tetrahedrally coordinated zinc (Zn) atom. The sequences N-terminal to the core contribute one α helix (H1), whereas the sequences C-terminal to the core contribute helices H8 and H9 (Stavridi et al. 2003).

On one side, the structure has a flat surface consisting of H1 and H2 and parts of H3, H4, H6, and H7. Stavridi et al. (2003) reports that most of the conserved residues of Mob family members map to parts of the flat surface formed by H2 and two loops, L1 and L2, adjacent to the N-terminus of H2. Loop L1 in human Mob protein goes from residues 46 to 51 and Leu47 and Pro48 are highly conserved since are needed to stabilize the structure of the loop. These results are confirmed in our analysis, with the exception of position 47 in Mob3 consensus sequence where a Pro is present. Moreover, Stavridi et al. (2003) reports that Glu51 is conserved only in Mob1 family. Figure 4 shows that Glu51 is conserved in Mob1 and Mob4 consensus sequences, while in Mob2 sequence is replaced by an isoleucine and in Mob3 by a glutamine.

The L2 loop, consisting of residue 128–142, presents several highly conserved amino acids involved in structural interaction, such as Pro133 and Pro141 and Phe132 and Phe140 that, together with Phe144 from H5, form hydrophobic interactions with each other and with Ala58 and Ile151 from H2 and H5, respectively. Figure 4 shows that all these positions are conserved, except for Mob3 where various non-conservative amino acid changes can be seen in the consensus sequence (Phe140→Glu140, Phe144→Val144, Ala58→ Tyr58). Moreover, the Mob3 consensus sequence is missing the amino acid in position 141.

Helix H2 has a large number of conserved residues, several of which have solvent exposed negatively charged side chains. While Stavridi et al. (2003) report that Asp52 is the only charged conserved residue in all Mob families, in our analysis we found that in Mob4 and Mob4a there is an amino acid conservative substitution Asp→Asn. Moreover, we observed that Glu55, that makes a hydrogen bond with Glu51, is conserved in Mob1, Mob2 and Mobp groups while Mob4 contains aspartate and the consensus sequence of Mob3 contains a valine. Asp63 interacts with His185, that is conserved in all Mob consensus sequences except for Mob3 that contains a lysine. Interestingly, Asp63 is conserved in all Mob4, Mobp and Mob1 classes, but it is replaced by a threonine in Mob2 and by a glutamine in Mob3.

Towards the C-terminal of helix H2 there is Asn69, the only polar residue other than tyrosines, that is conserved in all members of the Mob family. H2 also has several hydrophobic residues that are conserved to varying degrees in members of the Mob protein family: notably, Trp56 and Phe64, which should have buried side chains and participate in hydrophobic interactions that stabilize the protein fold, are conserved in all Mob consensus sequences.

A Zn binding site appears to be conserved in all Mob classes, with a peculiar exception in fungi. Considering human Mob1 protein as a reference, the Zn binding site is composed by Cys79 and Cys84 from loop connecting H3 to the first strand of the β hairpin and His161 and His166 from H5 (Stavridi et al. 2003). The presence of the Zn atom contribute to the stability of the structure by anchoring H3 to the C terminus of H5. As reported in Mrkobrada et al. (2006) most of the yeast genes previously described as Mob2A apparently lack the Zn binding site, since the two cysteines are substituted with a valine and a tyrosine respectively, suggesting an alternative structural element for stability compensations. The consensus sequences alignment confirms these observations with the *S. cerevisiae* NP_116618 as the only Mob protein lacking the Zn binding site (Fig. 4). To make sure that this observation was not due to a consensus artefact, we analyzed the complete 192 Mob-like protein multiple alignment (see supplementary Figure 1S) and we found that essentially all the proteins analyzed contained a well conserved Zn binding site. The only exceptions, found in *M. musculus* XP_001000051, *S. purpuratus* XP_001185390 and *M. mulatta* XP_001108825, are probably due to bad quality sequences producing an unreliable alignment in the region that contains His161 and His166.

## Biological Roles of Mob Proteins and Conserved Signaling Pathways

### Cell cycle progression and cytokinesis

**Figure 5.**
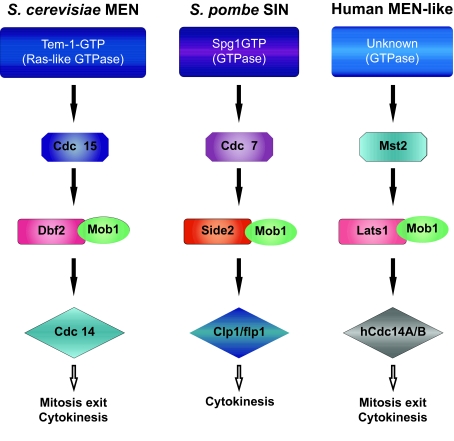
Components of the mitotic exit network (MEN) and septation initiation network (SIN) in yeasts *(Saccharomyces cerevisiae* and *Schizosaccharomyces pombe*), and of the MEN-like network in human cells. Exit from mitosis and co-ordination with cytokinesis is driven through a GTPase signaling network, where Mob1p is an essential regulator of the localization and activity of Dbf2 and Dbf2-like (Sid2 and Lats1) protein kinase. The network promotes the inactivation of the mitotic Cdk1-cyclin B complex and drives mitotic exit by leading to the release of the Cdc14p phosphatase from the nucleolus and its subsequent activation during anaphase.

The involvement of Mob proteins in cell proliferation was first suggested by Luca and Winey in 1998. They demonstrated that Mob1 is an essential yeast gene required for the completion of mitosis and maintenance of ploidy, as yeast Mob1 mutations resulted in a late nuclear division arrest at restrictive temperature. Following studies better elucidated the biological role of this protein in budding and fission yeasts. In *Saccharomyces cerevisiae* Mob1p is an essential regulator of the localization and activity of Dbf2 protein kinase, a component of the mitotic exit network (MEN). MEN is a GTPase driven signaling network that co-ordinates exit from mitosis with cytokinesis (Fig. 5). It promotes the inactivation of the mitotic Cdk1-cyclin B complex and drives mitotic exit by leading to the release from the nucleolus and subsequent activation of the Cdc14p phosphatase during anaphase (Luca et al. 2001; Stegmeier and Amon, 2004). Although inactivation of Cdk1-cyclin B complex is required for cytokinesis, the MEN was shown to be essential for cytokinesis, and in particular for actomyosin ring contraction and septum deposition, also independently of its role in mitotic exit. In fact, when MEN function is abrogated in conditions where mitotic exit is allowed by artificial suppression of mitotic CDK activity cytokinesis does not take place (Shou et al. 1999; Lippincott et al. 2001; Park et al. 2003).

In *S. pombe* cytokinesis is regulated by a signaling cascade termed the septation initiation network (SIN). It is organized similarly to the MEN but is not involved in mitotic exit (reviewed by Simanis, 2003; Krapp et al. 2004; Wolfe and Gould, 2005). In *S. pombe* Mob1 is part of the SIN and interacts with Sid2, the ortholog of *S. cereviasae* Dbf2, regulating its localization and kinase activity. Nevertheless, how Mob proteins can regulate kinase activity is still under investigation. By analyzing the NMR or X-ray crystal structures of *S. cerevisiae*, *X. laevis* and human Mob1p, it has been proposed that Mob proteins may regulate their target kinases through electrostatic interaction mediated by conserved charged surfaces. It seems that the negatively charged surface on MOB proteins interacts directly with the positively charged basic—hydrophobic N terminus of their target kinases Dbf2/Sid2, inducing a conformational change which enable the upstream kinase Cdc15/Cdc7 to phosphorilate and thereby stimulate DBf2/Sid2 activity. In this regard, MOB proteins may functionally resemble cyclins (Stavridi et al. 2003; Ponchon et al. 2004; Mrkobrada et al. 2006). However, yeast Mob1 proteins do not function solely as activators of Dbf2/Sid2, but are also required for Dbf2/Sid2 localization to activation sites (Frenz et al. 2000; Lee et al. 2001). It has been extensively reported that, in agreement with their functions in mitosis exit and cytokinesis, Dbf2/Sid2-Mob1 complexes localize to the spindle pole body (SPB) in anaphase and move to the division site in late mitosis (Stegmeier and Amon, 2004). Nevertheless, it must be underlined that the function of Dbf2/Sid2 in cytokinesis and how this complex ultimately leads to release of Cdc14 from the nucleolus during mitotic exit remain unclear. One reason is that, while the components of MEN that act upstream of Dbf2-Mob1 have been characterized, the molecular substrates for Dbf2-Mob1 have yet to be identified. At this regards Mah et al. (2005) determined that Dbf2-Mob1 preferentially phosphorylates serine over threonine and required an arginine three residues upstream of the phosphorilated serine in its substrate (RXXS motif).

Recent findings suggest also an involvement of MEN-Mob1p in coordinating chromosome segregation and/or spindle integrity with mitotic exit and cytokinesis via regulation of chromosome passenger proteins. Mob1p has been demonstrated to be essential for maintaining the localization of Aurora, INCENP, and Survivin chromosomal passenger proteins on anaphase spindles and for dissociating Aurora from the kinetochore region (Stoepel et al. 2005). Consistent with these functions, the MEN protein kinase complex Mob1p-Dbf2p localizes to mitotic nuclei and partially co-localizes with Cdc14p and kinetochore proteins.

Overall the available data in yeast indicates an essential role of Mob1p in cell cycle progression, through the interaction with Dbf2/Sid2 protein kinases and reveals an essential temporal and spatial regulation of Mob1 activity.

MEN components are conserved through evolution and in particular Mob1 and Dbf2-related proteins have been found in both animal (Stavridi et al. 2003; Ponchon et al. 2004; Devroe et al. 2004) and plant cells (Van Damme et al. 2004; Citterio et al. 2005, 2006), suggesting that their role in controlling cell cycle progression might be conserved in higher eukaryotes. The demonstration that animal Dbf2 homologous proteins NDR (nuclear Dbf2-related) genetically and physically interact with Mob1-related proteins (Bothos et al. 2005; Hammarton et al. 2005; He et al. 2005; Lay et al. 2005) and the determination of the yeast, human and *X. laevis* Mob protein structures, suggest that Mob proteins act as kinase activating subunits also in higher eukaryotes.

Nevertheless the biological roles of MOB proteins are still to be understood. In higher eukaryotes multiple MOB members are involved in multiple pathways. To date two probably distinct signaling networks, namely MEN and HIPPO (Bothos et al. 2005; Edgar, 2006), controlling cell proliferation and involving Mob1-like proteins have been recently proposed in *Drosophyla* and mammalian cells (see Hergovich et al. 2006). HIPPO pathway has been described in flies where participates to the control of tissue growth. This network includes cell cycle and cell death regulators, such as Hippo (Hpo), Salvador (Sav), Lats/Warts (dNDRs), Mats (Mob as tumor suppressor, dMob1) and Yorkie (Yki) factors (reviewed by Edgar, 2006). All components of the HIPPO pathway are well conserved in mammals and researchers have hypothesized that they share a similar function in humans. The complex Lats-Mob1A was also indicated as a component of the uncharacterized MEN network in higher eukaryotes. Bothos et al. (2005) have demonstrated that, similarly to ScMob1, hMob1A interacts and co-localizes with Lats1 at the centrosomes and midbody and that the suppression of Lats1 or hMob1A extends telophase but not other phases of mitosis. On the basis of the identification of evolutionary conserved MEN components the authors suggested the presence of a MEN conserved pathway in higher eukaryotes (Fig. 5). Given the complexity of the interactions it is possible that different isoforms of hMob1A and Lats belong to specific network and/or that the activation of different pathways is organism, tissue and/or cellular context dependent. Also the subcellular localization of the hMob1A-Lats1 complex is likely determinant for Lats1 activation and function. Hergovich et al. (2006) demonstrated that the membrane-targeting of hMob1A results in a significant increase of Lats1 activity in mammalian cells, while the simple co-expression of Lats1 with hMob1A does not elevate Lats1 kinase activity. On the other hand, the presence of a MEN pathway in higher eukaryotes is also suggested by the study of Mob1 proteins in plants (Citterio et al. 2006). *Medicago sativa* Mob1 proteins are mostly expressed in actively proliferating tissues and their localization pattern shares many features with that of yeast, despite the differences in mitotic entry and progression between the two organisms. The subcellular localization of MsMob1-like proteins is cell cycle-regulated. In alfalfa cells, Mob1 proteins forms grains in the cytoplasm from which fibrillar structures radiate in all directions, preferentially toward the cell mid-plane. These grains could likely correspond to sites in which microtubules are reorganized during cell cycle progression, the yeast SPBs, and barely detectable in G_1_ and S cells, whereas become evident in G_2_, forming clusters around the nucleus. In mitosis, they preferentially localize at the two opposite cellular poles. Differently from yeast, in alfalfa cells undefined Mob1 fibrillar structures are formed. In addition, during pre-prophase Mob1-like proteins mark the inner border of the cell wall in correspondence with the outer parts of the pre-prophase band, and in cytokinesis besides the progressive labeling of the septum, forms fibrillar structures, that partially co-localize with phragmoplast microtubules and partially form an aster, radiating from the growing septum poles.

**Figure 6.**
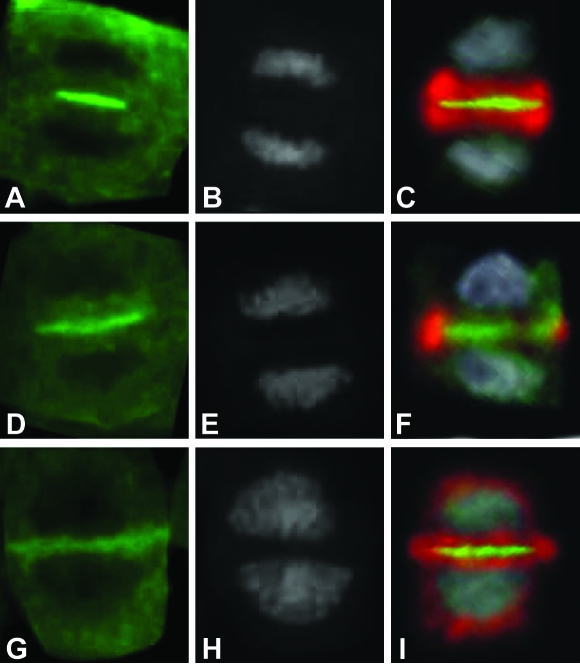
Results of the simultaneous immunolocalization of Mob1-like proteins (green fluorescence, Panels **A, D** and **G**) and alpha tubulin (red fluorescence, Panels **C, F** and **I**) in alfalfa cells during three successive stages of cytokinesis (the yellow fluorescence represents tubulin and Mob1-like protein co-localization). DNA was also stained with DAPI (gray signal, **B, E** and **H**). Mob1-like proteins are localized to the cell division midplane during cytokinesis, marking the progressive formation of the phragmoplast (for additional information, see Citterio et al. 2006).

Overall the results collected so far in plants indicate that Mob1-like proteins are involved in cell proliferation, are expressed in a cell cycle-dependent manner and are localized to the cell division midplane during cytokinesis, marking the progressive formation of the phragmoplast, as shown in Figure 6.

An interesting possibility is that Mob1-like proteins participate to the orientation of cell plate during cytokinesis, interacting with cytoskeletal structures and conjugating the determination of division site, marked by pre-prophase band before the onset of mitosis, with the septum formation (Citterio et al. 2006). Nevertheless the expression of MsMob1 could not rescue the lethality of the yeast mob1 mutant. This inability can be attributed to several reasons and does not rule out that the two genes do encode functional homologs. It is possible that MsMob1 does not bind efficiently to budding yeast Dbf2, thus explaining the lack of cross-complementation. Importantly, amino acid residues of ScMob1, such as Thr105, Leu196 and Cys221, that are changed in mob1 mutant alleles and presumably crucial for Mob1 function (Luca and Winey, 1998; Stavridi et al. 2003), are replaced in a non-conservative way in the MsMob1 primary sequence, suggesting that in spite of their high degree of similarity the two proteins might have substantially diverged and that the interaction of Mob1 proteins with their effectors may be species-specific.

On the whole, the available data strongly suggest that in higher eukaryotes as in yeast Mob1 members of MOB family play a role in the control of cell proliferation, through the regulation of NDR activity and localization. However further experiments are needed to better understand the roles of the single Mob1-like genes in each type of organism and tissue.

## Apoptosis and Programmed Cell Death

In a multicellular organism, the maintenance and surveillance of organ size is essential. Any imbalance in the relationship between cell size, cell proliferation and cell death must be prevented to allow proper organ development and to maintain the integrity of organ tissue over time. Failure to coordinate the creation of new cells (proliferation) and the elimination of excess ones (by apoptosis) can lead to diseases (Green and Evan, 2002). Mob proteins are involved in the control of cell death and its coordination with cell proliferation, being direct co-activators of NDR (nuclear Dbf2-related) kinases.

**Figure 7.**
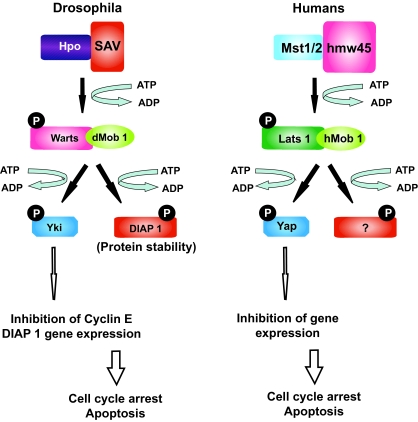
The HIPPO (hpo)-Large tumor suppressor (Lats) pathway validated in *Drosophila melanogaster* and its similar pathway recently postulated in mammals. The network involves Hippo (Hpo), Salvador (Sav), Lats1/Warts (dNDRs), Mats (Mob as tumor suppressor, dMob1) and Yorkie (Yki) factors, and participates to the control of tissue growth by regulating cell cycle arrest and cell death. In *Drosophila* Hpo interacts with Sav, which acts as a scaffold protein, and phosphorilates Warts-Mats. Activated Warts can negatively regulate the transcription of cell cycle and cell death regulators such as cyclin E and the apoptosis inhibitor DIAP1, through the phosphorilation of the non-DNA binding transcriptional co-activator Yorkie. All components of the HIPPO pathway are well conserved in mammals and they have a similar function in humans since Lats1 (Warts), Mob1A (Mats), MST2 (Hippo) and Yap (Yorkie) genes can all functionally rescue their correspondent *Drosophila* mutants.

Recent advances using *D. melanogaster* lead to the identification of a pathway that participates in the control of tissue growth (Harvey et al. 2003; Jia et al. 2003; Pantalacci et al. 2003; He et al. 2005; Huang et al. 2005). The control of cell death and proliferation by the Hippo (hpo)-Large tumor suppressor (Lats) pathway was demonstrated and a similar pathway was also postulated in mammals (Fig. 7). In *Drosophila*, four factors that induce tissue overgrowth without affecting pattern formation were identified: Sav, Hpo, Lats and dMob1/Mats (reviewed by Hergovich et al. 2006). Loss of any of these factors results in tissue overgrowth which is associated with increased cell proliferation and decreased cell death, indicating that Sav, Hpo, Lats and dMob1 all function as tumour suppressors. Genetic and biochemical independent studies indicate that Hpo interacts with Sav, which acts as a scaffold protein, and phosphorilates Warts-Mats. The association of Mats with Warts is essential in this regulatory process, as flies that carry mutation in Mats are unable to control tissue growth, despite having a functional Warts. Activated Warts has been proposed to negatively regulate the transcription of cell cycle and cell death regulators. Interestingly, the tissue overgrowth phenotype in *Drosophila* is accompanied by elevated levels of an important regulator of S-phase entry (i.e. cyclin E) and Diap1 (*Drosophila* inhibitor of apoptosis protein-1), an inhibitor of apoptosis. Moreover, *Drosophila* Salvador (Sav) interacts biochemically with Hpo, thereby facilitating the activation of Lats by phosphorylation (Harvey et al. 2003; Pantalacci et al. 2003; Wu et al. 2003). The activated Lats-dMob1 (*Drosophila* Mps1-one binder-1) complex then inactivates Yorkie (Yki) by phosphorylation (Huang et al. 2005). Phosphor-ylated Yki can not stimulate the expression of cyclin E and Diap1, which results in decreased cell proliferation (low cyclin E) and increased cell death (low Diap1).

It is worth noting that the association of dMob1 with Lats is essential in this regulatory process, as flies that carry mutations in dMob1 are unable to control tissue growth, despite having a functional Lats (Lai et al. 2005). Therefore, Lats that is phosphorylated by Hpo needs to bind to its co-activator dMob1 to properly coordinate cell death and proliferation (Fig. 7). As a matter of fact, cells that carry mutations in Hpo, Sav, Lats and dMob1 show an accelerated proliferation, but maintain a normal size. As a consequence, loss of these genes must stimulate cell growth and reduce cell death.

In mammals, a similar pathway was postulated (Fig. 7). Several human orthologs of the Hpo–Sav–Lats–dMob1–Yki pathway have emerged as putative tumour suppressors (Tapon et al. 2002; Lai et al. 2005; Takahashi et al. 2005; Jimenez-Velasco et al. 2005). Human mammalian sterile 20-like kinase (MST1/2) associates with hWW45 (the human ortholog of Sav) and activates LATS1/2 by phosphorylation (Chan et al. 2005). The LATS–hMOB1 complex then potentially activates specific gene expression programs through YES-associated protein (YAP). Similar to large tumour suppressor (Lats) in invertebrates, several findings point to LATS functioning as a tumour suppressor in mammals (St John et al. 1999; Hisaoka et al. 2002; Takahashi et al. 2005; Jimenez-Velasco et al. 2005). The significance of functional conservation is further strengthened by the fact that human MST2, hMOB1A and LATS1 can rescue the tissue-overgrowth phenotype of Hpo, dMob1/Mats and Lats mutants in *D. melanogaster* (Wu et al. 2003; Lai et al. 2005). Moreover HIPPO components, including Mob1A are mutated in mammalian tumors.

Overall, LATS seems to be a tumour-suppressor protein that is conserved in flies and humans, whereas the roles of mammalian NDR1/2 and their co-activators MOBs are yet to be fully established. Existing findings indicate that mammalian NDR1/2 could function as protooncogenes (Hergovich et al. 2006).

Like in animals, also in plants specific cell types undergo programmed cell death (PCD) as part of their developmental and differentiation program (Vaux and Korsmeyer, 1999). From embryogenesis to fertilization, cell and tissue death is an integral part of plant development and morphogenesis as well as a response to the environment (Barlow, 1982; Buckner et al. 1988). Even though the cellular deterioration patterns described in plant tissues are in some cases similar to those observed in animal tissues, little is known of the mechanisms that control PCD in plants (Pennell and Lamb, 1997; Allen et al. 1998; Vaux and Korsmeyer, 1999). In angiosperms, PCD occurs late in the degenerative stage of the reproductive phase in both anther and pistil (Wu and Cheung, 2000). Production of functional male gametes depends largely on the deterioration and death of the anther tapetum, whose main functions appear to be the nurturing of microspores with cortical surface molecules and allowing pollen dispersion at maturity. The pathway of female gametogenesis frequently begins with the death of all but one reduced megaspores, while surrounding nucellar cells degenerate in concert with embryo sac expansion (Reiser and Fisher, 1993; McCormick, 1993; Barcaccia et al. 2003).

**Figure 8.**
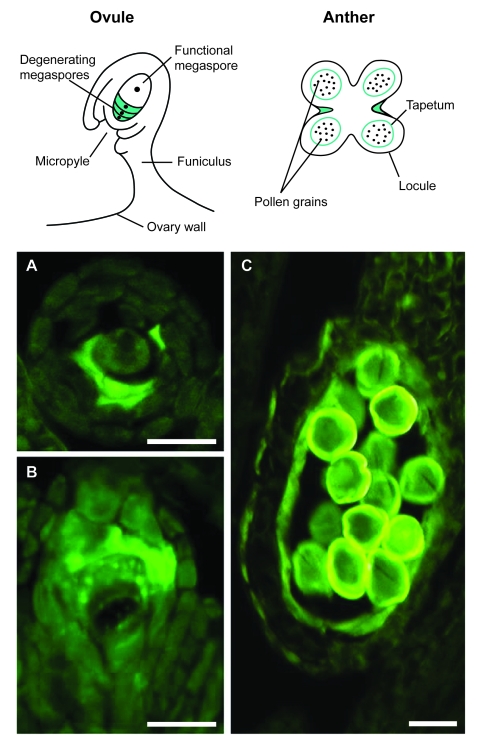
Mob1-like expression patterns in plant reproductive tissues, with particular reference to alfalfa (*Medicago sativa* L.). The cartoons show spores and cells that most prominently undergo programmed cell death (PCD) in ovules and anthers (adapted from Wu and Cheung, 2000). In ovules at the end of sporogenesis, proteins are mainly visualized in the reduced megaspores undergoing PCD or the remnants of degenerated megaspores (**A, B**), whereas in anthers, proteins are specifically found at the end of gametogenesis in tapetum cells naturally undergoing PCD to allow pollen grain dispersal (**C**). Bar: 20 μm (for experimental details, see Citterio et al. 2005).

Mob1 may be a component of a complex of proteins with multiple functions, not only involved in cytokinesis, cell proliferation and morphogenesis, but also operatively associated with cell death. Database searches revealed that MOB domain (pfam03637) can be combined in complex proteins with elements of the NB-ARC domain (pfam00931), a signaling motif shared by animal cell death gene regulators. Proteins containing a highly conserved Mob1 domain include also receptors for ubiquitination targets (F-Box), Ser/Thr and Tyr kinases as well as CBL (Calcineurin B-Like)-interacting kinases which may be implicated in either cell proliferation or cell death. The possible involvement of Mob1 proteins in PCD is also supported by our recent analysis of Mob1-like expression in alfalfa reproductive tissues (Fig. 8). In the ovules during gametogenesis, both transcripts and proteins were mainly visualized in the reduced megaspores undergoing PCD or in the remnants of degenerated megaspores, whereas in the anthers, Mob1-like gene products were specifically found at the end of gametogenesis in tapetum cells naturally undergoing PCD to allow pollen grain dispersal (Citterio et al. 2005). Moreover, localization of MOB-domain containing proteins was also documented in alfalfa meristematic tissues of the plant roots. It is known that the root cap consists in living parenchyma cells derived continuously from the apical meristem and programmed to die: as new cells are produced in the interior, those on the root periphery are shed in an orderly manner. Hybridization signals were detected in a thin cell layer of the root apex where meristematic root tip cells divide and differentiate in root cap. Such finding further supports the concept that Mob proteins are related to the onset of programmed cell death in plants (Citterio et al. 2006).

Further experiments will help clarifying the function of Mob1-like proteins in both cell proliferation and PCD. The challenge will be to dissect the roles of each Mob1-like gene in different tissues. The production and exploitation of specific antibodies against each of the Mob1-like gene products encoded by a specific member of the MOB family should aid in determining whether a multi-domain protein component with distinct functions is operative during cell proliferation and PCD.

## Cell Polarity and Morphogenesis

The MOB2-NDR proteins are central factors of the RAM (Regulation of Ace2 Activity and Morphogenesis) network in cell separation and polarity establishment. In this section we will briefly review on the progress made so far on the elucidation of the role played by MOB proteins and NDR kinases in regulating cell morphology in co-ordination with the mitotic exit. Co-ordinating asymmetric cell division, and establishment and maintenance of cell polarity are essential processes in growth and differentiation. Polarized morphogenesis is necessary for proper functioning of specific cell types such as neurons, epithelial cells, plant root hairs and pollen tubes and fungal hyphae and its core elements are substantially conserved across eukaryotes. Cell intrinsic polarity is established early during cell division and factors governing cell separation and cell polarity are tightly controlled and co-ordinated.

**Figure 9.**
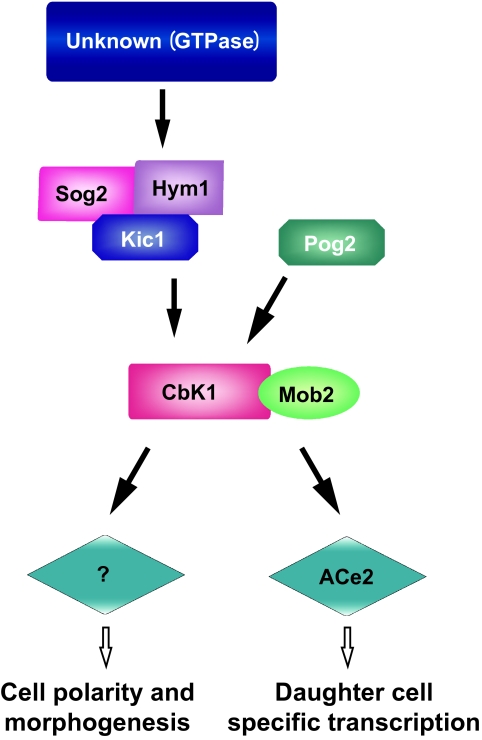
Schematic representation of the *S. cerevisiae* RAM network. The protein kinase Kic1 associates with the proteins Sog2 and Hym1 to form a complex necessary for proper localization and function of Cbk1. In analogy to its counterpart Cdc15 in the MEN network, Kic1 likely activates Cbk1 directly. Pag1 interacts with Kic1 and Cbk1 facilitating its activation. Cbk1 requires the interaction with Mob2 for activation and to regulate the transcription factor Ace2, essential for cell separation to occur, through the transcription of genes involved in cell wall synthesis in a daughter cell specific way. The Cbk-Mob2 complex also regulates polarized growth of cells, proper bud site selection and formation of mating projections through a largely uncharacterized Ace2 independent pathway.

Studies carried out on yeast, have led to the identification of the so-called RAM network of proteins as a central element involved in the early phases of polar morphogenesis during cell separation (Nelson et al. 2003). The core components of the yeast RAM network are the LATS/NDR kinase CBK1p and its upstream regulator MOB2p, which play a dual role in controlling mother-daughter cell separation and establishment of cell polarity. Cell separation in yeast relies on the daughter cell specific expression of genes necessary for septum degradation, shown to be dependent on the specific localization and activation of the ACE2 transcription factor in the daughter cell nucleus together with MOB2p and CBK1p at the end of mitosis (Colman-Lerner et al. 2001; Weiss et al. 2002). Loss of function strains mob2pΔ and cbk1pΔ as well as ace2pΔ show defects in the cell separation process resulting in clumps of cells. However, interestingly, the mob2pΔ and cbk1pΔ cells, but not the ace2pΔ, display loss of polar growth suggesting that the MOB2p-CBK1p complex regulates cell morphology through a specific pathway that is independent from Ace2 activity (Weiss et al. 2002; Nelson et al. 2003). Cells deleted for either CBK1 or MOB2 or expressing a catalytically inactive form of Cbk1p in *S. cerevisiae* (Racki et al. 2000; Bidlingmaier et al. 2001; Colman-Lerner et al. 2001; Weiss et al. 2002) or lacking CBK1 and MOB2 orthologs in *S. pombe* (Verde et al. 1998; Hou et al. 2003) are round and lack axial polarization, proper bud selection and mating projections. In addition cells lacking a functional MOB2p-CBK1p machinery display multiple sites of bud selection and growth suggesting a general role for these proteins in determining early events for cell polarity establishment (Nelson et al. 2003). A schematic representation of the *S. cerevisiae* RAM network is reported in Figure 9.

Based on genetic and biochemical studies in yeast, MOB2p–CBK1p activity is placed downstream of and dependent on the functional presence of the other RAM proteins KIC1p, HIM1p, TAO3p and SOG2p with KIC1p, HIM1p and SOG2p forming a functional complex required for MOB2p–CBK1p phosphorylation and activation (Nelson et al. 2003). The KIC1p kinase, the second kinase of the RAM signaling network together with CBK1, displays significant sequence similarity to the MEN kinase Cdc15p, involved in the activation of the MEN MOB1p–DBF2p kinase complex directly (Mah et al. 2001), and it has been shown to activate Mob2p-Cbk1p for regulating Ace2p and cellular morphogenesis (Nelson et al. 2003). These data suggest the conservation of the core interaction of MOB and NDR proteins in both MEN and RAM networks and of their mode of regulation by immediate upstream factors. Furthermore, the role of the MOB2-NDR complex in establishing cell polarity seems to be conserved throughout eukaryotes, since loss of function of CBK1/ORB6-related NDR kinases leads to defects in cell axialization and cell spreading and/or branching also in *Drosophila*, *C. elegans* and in mammalian cells. However, while loss of CBK1 function in yeast leads to a failure in axialization and bud selection of cells (Racki et al. 2000; Bidlingmaier et al. 2001; Colman-Lerner et al. 2001; Du and Novick, 2002; Weiss et al. 2002; Nelson et al. 2003) the inactivation of the *Drosophila* NDR encoding gene tricornered (trc) leads to split epidermal hairs and bristles (Geng et al. 2000) and augmented dendritic branching (Emoto et al. 2004). Similar defects in dendritic branching are observed in the presence of mutations of the *C. elegans* NDR encoding gene Sax1 (Zallen et al. 2000) suggesting a negative role exerted by NDR kinases in the control of cell axialization and branching in higher eukaryotes opposite to the positive role played by the MOB2p-CBK1p complex of yeast. Hyperpolarization instead of loss of polarization has also been shown following systematic mutagenesis of components of the RAM network in the pathogenic fungus *Cryptococcus neoformans* (Walton et al. 2006). This was observed in the presence of substantial conservation of subcellular localization and protein-protein interactions between MOB2p and CBK1 homologs and upstream components (Walton et al. 2006), further suggesting a general conservation of the central role of the MOB2p-CBK1p/NDR complex in directing cell polarity in eukaryotes, but pointing to a probable divergence of downstream components leading to opposite cell polarity phenotypes. This may reflect different mechanisms of cell shape control via the re-organization of the cytoskeleton through assembly of actin cables, controlled for example in yeast by formin (Burns et al. 1994; Evangelista et al. 2002), or via alternative systems. Interestingly, the MOB2p-CBK1p complex seems to regulate cell polarity through a mechanism that is at least partly independent from the actin cables assembly since in RAM mutants actin organization has been reported to be not substantially affected (Weiss et al. 2002; Nelson et al. 2003). In addition MOB2 or CBK1 mutations result in additive phenotypes when combined with mutations affecting the formin encoding gene Bni1 (Du and Novick, 2002; Weiss et al. 2002; Nelson et al. 2003). This together with the finding that Cbk1p has been shown to bind Sec2p, a guanine nucleotide exchange factor involved in vesicle transport and exocytosis (Racki et al. 2000), have lead to the hypothesis that the RAM network may act in cell polarity through regulation of vesicle transport (Terbush et al. 1996; Lipschutz and Mostov, 2002). On the contrary, the *Drosophila* Trc gene functions altering actin and microtubule organization (He et al. 2005) and has been placed on the same genetic pathway of RhoA GTPase since loss of Trc function and expression of a dominant negative form of RhoA result in similar non additive phenotypes (He et al. 2005). Rho GTPases are well known players in cell polarity establishment through the regulation of actin dynamics, however even though it has been suggested that they may be downstream components of NDR kinases in *Drosophila* (He et al. 2005) and in *C. elegans* (Zallen et al. 2000), definitive biochemical evidence is needed to fully clarify their exact hierarchical relationships. In fact, it cannot be excluded that the MOB-NDR complex may be a downstream component of Rho GTPases, also considering the similarity of NDR kinases with Rho kinases, the immediate downstream components of Rho signaling.

## General Discussion and Concluding Remarks

The MOB family includes a group of cell cycle-associated, non-catalytic proteins highly conserved in eukaryotes, whose founding members are implicated in mitotic exit and co-ordination of cell cycle progression with cell polarity and morphogenesis (Luca et al. 2001; Stegmeier et al. 2002; Nelson et al. 2003).

An HMM search for Mob-like domain containing proteins in 43 completed and ongoing eukaryotic genomes highlights the universal distribution of this protein family in the so-far sequenced organisms, suggesting its prominent biological function. The phylogenetic analysis reveals five distinct classes of the MOB domain, resulting in the necessity of a reassessment of the relationship existing among the proteins found in different taxa. As an example, in our analysis the founding member ScMob1 does not cluster within the Mob1 group, as previously reported in various papers (Stavridi et al. 2003; Mrkobrada et al. 2006).

Analysis on Mob domain distribution reveals a progressive expansion of this family from unicellular to multicellular organisms, reaching the highest number in mammals. Moreover, phylogenetic analysis shows that the Mob4 genes form a peculiar class of the invertebrata taxa, that underwent an expansion in vertebrata giving origin to Mob4a and Mob4b classes. Plant Mob genes appear to have evolved from a single ancestor, most likely due to the loss of one or more genes during the early stage of Viridiplantae evolutionary history. Finally Mob1, Mob2 and Mob3 classes are widespread among almost all analyzed organisms. Mob3 class is the most divergent one, suggesting a possible different function for the genes belonging to this class. Mob2 class, compared to the other Mob classes, presents a lower gene identity percentage homogeneity, revealing the possible presence of other subgroups belonging to this class.

Different distribution and phylogenetic relationship among genes of the same organism and/or different organisms suggest that the Mob gene family evolves under a birth-and-death evolution model (Nei and Rooney, 2005).

Two distinct Mob proteins, Mob1 and Mob2, are known in fungi, while an expansion in metazoans gives rise to six (seven) in human, four in *D. melanogaster*, and four in *C. elegans* (Mrkobrada et al. 2006). Mob1 proteins have been demonstrated to be important for both mitosis completion and cell plate formation in yeast (Luca and Winey, 1998). Moreover, the Mob1-related proteins Mob2 physically associate with specific kinases throughout the cell cycle, being required and periodically activated in yeast to promote polarized growth (Weiss et al. 2002). Mob1-like proteins have been also found in animals (Stavridi et al. 2003; Ponchon et al. 2004; Devroe et al. 2004). Plant genomes such as alfalfa, rice and *Arabidopsis* contain uncharacterized Mob1-related genes (Van Damme et al. 2004; Citterio et al. 2005; 2006). Although there are data to suggest that Mob1 proteins act as kinase activating subunits in higher eukaryotes, their function remains to be proved. Present findings suggest that animal and yeast Mob1 may have similar functions.

That Mob1 proteins play a crucial role in cytokinesis has been demonstrated in yeast (Luca and Winey, 1998). The study of a spontaneous lethal mutation in a *Drosophila* Mob1 gene has recently implicated the MOB-domain containing proteins in the control of animal cell proliferation and apoptosis (Lai et al. 2005). Moreover, the identification of the animal Dbf2 homologous proteins NDR (Nuclear Dbf2-Related) interacting with Mob1-related proteins, and the determination of the human and *Henopus laevis* Mob protein tridimensional structures, may mean that Mob proteins act as kinase activating subunits even in higher eukaryotes. The functional co-dependence and cell cycle regulation of the Mob and Dbf2-like proteins is reminiscent of how cyclins bind and regulate cyclin-dependant kinases (Morgan, 1996; Mah et al. 2001).

MOB-domain containing proteins represent essential regulators of the localization and activity of nuclear Dbf2-related (NDR) protein kinases, components of the mitotic exit network (MEN) in yeast and MEN-like in human. Several lines of research in mammals are now in progress to define the precise roles of NDR interactors, particularly the regulation of Mob activators and MST kinases. A general regulation scheme at the molecular level, probably valid for all NDR family members, has recently been established (see Hergovich et al. 2006). The binding of the co-activator MOB-domain containing proteins to the N terminus of NDR kinases seems crucial for activation and function. It is known that Mob proteins interact with NDR-type kinases by binding a conserved stretch of primary sequence at their N-terminal regulatory domain. The interaction of Mob proteins with the NTR activation site is a conserved feature of all members of the NDR kinase family that have been tested so far in yeasts, flies and humans. Interestingly, Mob proteins do not function solely as co-activators of NDR kinases, but are also required for the localization of yeast NDR kinases. As a matter of fact, members of the NDR family are essential genes in both uni- and multicellular organisms. Dbf2p and Sid2p regulate mitotic exit and cytokinesis in yeasts, and their counterparts in mammals and plants could also have a similar role.

Recent advances lead to the identification of the Hippo signaling pathway that controls the coordination of apoptosis and cell proliferation, and tissue growth in *D. melanogaster* (see Hergovich et al. 2006). The association of Mob1p with Lats (Large tumor suppressor) is essential in this regulatory process since flies that carry mutations in dMob1 are unable to control tissue growth, despite having a functional Lats (Lai et al. 2005). Therefore, Lats that is phosphorylated by Hpo needs to bind to its co-activator dMob1 to properly coordinate cell death and proliferation. Interestingly, conserved key components of this pathway have been found to be mutated in human cancer samples, which indicates that a kinase network is probably conserved from flies to humans.

In plants, signaling mechanisms co-ordinate mitosis spatially and temporarily with cytokinesis to ensure integrity of genetic transfer during the cell cycle (Guertin et al. 2002), and important genes required for cytokinesis have recently been discovered. The involvement of plant Mob genes in cell cycle control is supported by recent data collected in *Arabidopsis* and *Medicago sativa* (Van Damme et al. 2004; Citterio et al. 2006). For instance, in *Arabidopsis* several putative cell cycle associated components (e.g. Mob1-like proteins) were targeted to the cell division plane and to the nucleus, suggesting that this organelle operates as a coordinating hub for cytokinesis (Van Damme et al. 2004). Moreover, in *M. sativa* Mob1-like proteins were proven to appear during late telophase and to localize across the entire cell division midplane, thus marking the progressive formation of the phragmoplast (Citterio et al. 2006). Nevertheless, the key role of MOB-domain containing proteins in plants is still poorly understood.

The greater amount of Mob1-like proteins in proliferating than in non-proliferating tissues, together with their cell cycle-regulated subcellular localization and their presence at the cleavage site suggest that these proteins may have a function in cell division similar to that of yeast Mob1 essential for mitotic exit and septum formation. In yeast, the spindle pole body operates as a signaling center during cytokinesis (Simanis, 2003). MEN/SIN regulators such as Sid kinases and Dbf2/Mob1 temporarily associate with the spindle pole body at some point in the cell cycle. For instance, in *S. cerevisiae*, Mob1 mobilizes to the spindle pole body (SPB) at anaphase and localizes to the bud neck, the future site for cell division, during cytokinesis (Hou et al. 2003). In analogy to this function, centrosomes have been implicated in completing cytokinesis in animals and human cells (Doxsey, 2001). In higher plant cells, microtubules (MTs) show dynamic structural changes during cell cycle progression and play significant roles in cell morphogenesis (Hasezawa and Kumagai, 2002). In addition to the cortical microtubules that control the cell shape, the preprophase band (PPB) and the phragmoplast are other plant-specific structures which can be observed from late interphase to prophase, and from anaphase to telophase, respectively. How plant MT arrays reorganize during the cell cycle is an unanswered question. Plants lack conventional animal centrosomes and yeast SPBs seem to possess flexible centrosomes from which nucleating material disperses at different cell cycle stages (Chan et al. 2003).

Despite differences between plant and yeast in mitotic entry and progression, the localization pattern in plant cells of Mob proteins shares many features with yeast (Van Damme et al. 2004; Citterio et al. 2006). In plant cells, Mob1-like proteins form grains in the cytoplasm from which fibrillar structures radiate in all directions, preferentially toward the cell midplane. These grains likely correspond to sites in which microtubules are reorganized during cell cycle progression. Proteins, barely visible in G_1_ and S, are clearly seen in G_2_ forming a ring around the nucleus, whereas during mitosis they preferentially localize as punctuate clusters at the two opposite cellular poles. Differently from yeast, in plants cells undefined fibrillar structures are formed. In cytokinesis besides the progressive labeling of the septum, Mob1-like proteins form fibrillar structures that partially co-localize with phragmoplast microtubules and partially form an aster, radiating from the growing septum poles. An interesting possibility is that Mob1-like proteins participate in cell plate orientation during cytokinesis, interacting with cytoskeletal structures and coupling the establishment of the division site, marked by PPB before the onset of mitosis, with septum formation. The interaction between MTs and Mob1p is emphasized by the characterization of haploid mob1 yeast mutants, which display a complete increase in ploidy at permissive temperature, caused by cytokinetic defects (Luca et al. 2001). However, although it is well demonstrated that yeast Mob1 is essential for the exit from mitosis and for septum formation, its exact function is still to be known even in this simple organism. Mob1 has been proposed to activate the mitotic exit network acting as an activating subunit of the Dbf2 protein kinase (Stavridi et al. 2003). In animal cells Dbf2 homologs interacting with Mob1-like proteins have been discovered (Ponchon et al. 2004; Devroe et al. 2004), suggesting a conserved function between yeast and higher eukaryotes. Nevertheless, Dbf2 homologs have not yet been characterized in plants.

The control of cell proliferation and cell death are central points of ongoing research programs in cell cycle control of all eukaryotes and, particularly, in human diseases by using model organisms. Basic studies addressed to the understanding of the mitotic events and its alterations will be crucial for practical applications in cell biology and medicine.

## Supplementary Information

Table 1S. The ?rst column reports the organism name, the second one the gene code whereas the third column the Mob class. Finally the fourth column shows the code number used in the multiple alignment in Figure 1S.

Figure 1S. Multiple alignment of the 192 Mob-domain containing proteins. The label number refers the fourth column in Table 1S of the supplementary materials and corresponds to the gene code.

## Figures and Tables

**Table 1.** Summary of available data on Mob-domain containing proteins and its interacting NDR-type kinases (see footnotes for main References).

**Organism**	**Protein name**	**Accession**	**Description/Function**	**Group**	**Subcellular localization**	**Interacting kinases**
*Saccharomyces cerevisiae*^1^	Mob1p	NP_012160	Component of the MEN: regulates mitotic exit and cytokinesis	-	Spindle pole body and bud neck	Dbf2p-Dbf20p
	Mob2p	NP_116618	Component of the RAM signaling network: links cell morphology changes with cell cycle progression	-	Nucleus, cytoplasm and cortex	Cbk1p
*Schizosaccharomyces pombe*^2^	Mob1p	NP_595191	Component of the SIN: controls septum initiation and cytokinesis	-	Spindle pole body and mitotic septum	Sid2p
	Mob2p	NP_587851	Required for maintenance of cell polarity: coordinates cell morphogenesis with cell cycle progression	-	Mitotic septum	Orb6p
*Caenorhabditis elegans*^3^	-	NP_510184	F09A5.4c	-		
	-	NP_502248	F38H4.10	-		
	-	NP_498798	C30A5.3	3		
	-	NP_501179	T12B3.4	4		
*Drosophila melanogaster*^4^	dMob1	NP_729716	CG11711-PB. Mob1, isoform B	2		Trc (dNDR)/Warts (Lats)
	Mats	NP_651041	CG13852-PA. Mob as tumor suppressor	1		Trc (dNDR)/Warts (Lats)
	dMob3	NP_609364	CG4946-PA	4		-
	dMob4	NP_610229	CG3403-PA	3		-
*Homo sapiens*^5^	hMOB1	NP_775739	MOB-KL1A, MOB kinase activator-like 1A (MOB1A)	1	Nucleus, cytoplasm and membrane	LATS1/2 (low affinity for NDR1/2)
	MATS1	NP_060691	MOB-KL1B, MOB kinase activator-like 1B (MOB1B)	1	Centrosome, poles of mitotic spindle and midbody	LATS1
	hMOB2	NP_443731	HCCA2 protein	2	Nucleus, perinuclear region and cytoplasm	NDR1/2
	hMOB3A	NP_955776	PREI3, preimplantation protein (Phocein)	3	Perinuclear region, membrane	PP2A
	hMOB3B	NP_079037	MOB-KL2B, MOB kinase activator-like 2B	4b		
	hMOB3C	NP_958805	MOB-KL2C, MOB kinase activator-like 2C	4a		
	MOB-LAK	NP_570719	MOB-LAK, metal ion binding	4b	Intracellular	
*Arabidopsis thaliana*^6^	Mob1A	NP_199368	Similar to yeast Mob1p	p	Nucleus	
	Mob1B	NP_193640	Mob1-like domain containing protein	p		
	Mob2A	NP_197544	Similar to yeast Mob2p	p	Nucleus	
	Mob2B	NP_197543	Similar to yeast Mob2p	p	Fragmoplast	
*Medicago sativa*^7^	Mob1A	CAC41010	Similar to yeast Mob1p	p	Cytoplasm and cell plate	
	Mob1B	CAG25780	Similar to yeast Mob1p	p		
*Trypanosoma brucei*^8^	Mob1A	AAL10512	Mob1-1 essential for cytokinesis but not for mitotic exit	-	Cytoplasm	tbPK50 (functional homolog of Orb6)
	Mob1B	AAL10513	Cell cycle associated protein Mob1-2	-		

^1^Luca et al. (1998); Luca et al. (2001); Komarnitsky et al. (1998); Mah et al. (2001); Stegmeier et al. (2002); Weiss et al. (2002); Mah et al. (2005); Stoepel et al. (2005).

^2^Verde et al. (1998); Salimova et al. (2000); Hou et al. (2003; 2004).

^3^No references.

^4^Geng et al. (2000); He et al. (2005); Lai et al. (2005).

^5^Moreno et al. (2001); Bichsel et al. (2004); Devroe et al. (2004); Hergovich et al. (2005); Lai et al. (2005); Bothos et al. (2005); Hergovich et al. (2006).

^6^VanDamme et al. (2004); Barcaccia et al. (unpublished).

^7^Citterio et al. (2005); Citterio et al. (2006).

^8^Hammarton et al. (2005).
