# Supplementary figures and images for: Characterization and Evolution of the Cell Cycle-Associated Mob Domain-Containing Proteins in Eukaryotes
Source: Evol Bioinform Online. 2007 Aug 8;3:121–58. (PMC2684140)

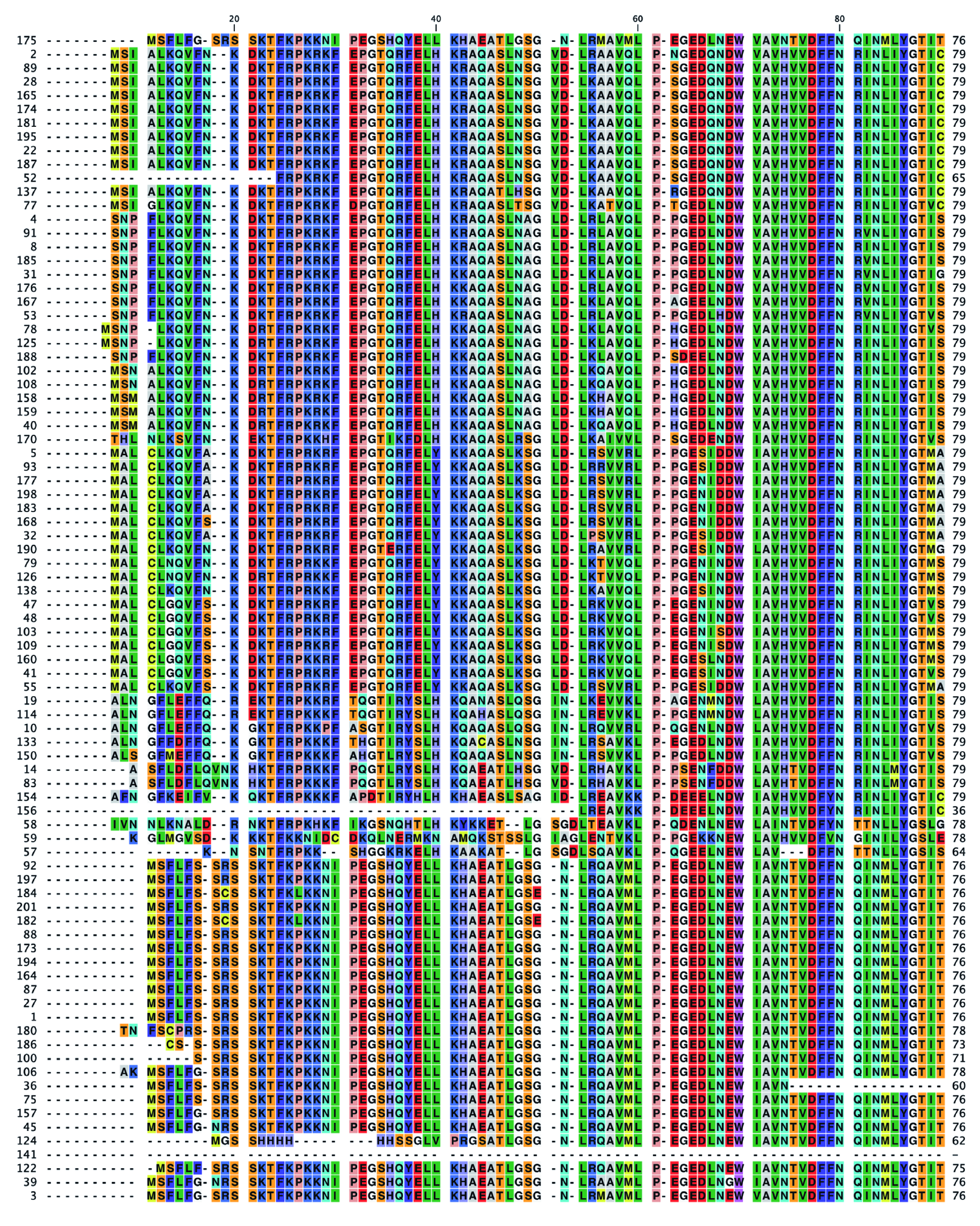

Supplement: Figure 1S. — Multiple alignment of the 192 Mob-domain containing proteins. The label number refers the fourth column in Table 1S of the supplementary materials and corresponds to the gene code. [file EBO-03-121-g00S1.tif]

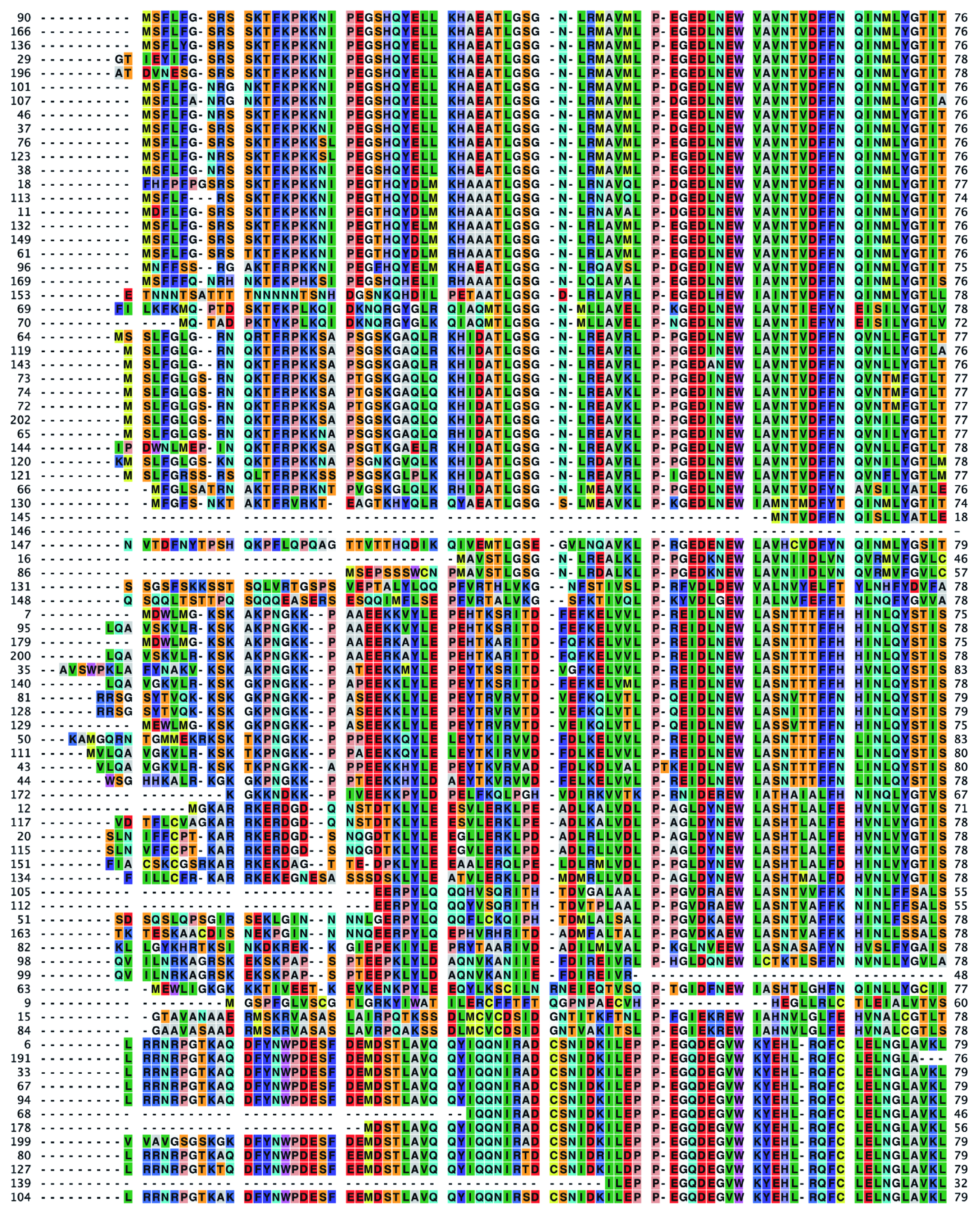

Supplement: Figure 1S. — Multiple alignment of the 192 Mob-domain containing proteins. The label number refers the fourth column in Table 1S of the supplementary materials and corresponds to the gene code. [file EBO-03-121-g00S2.tif]

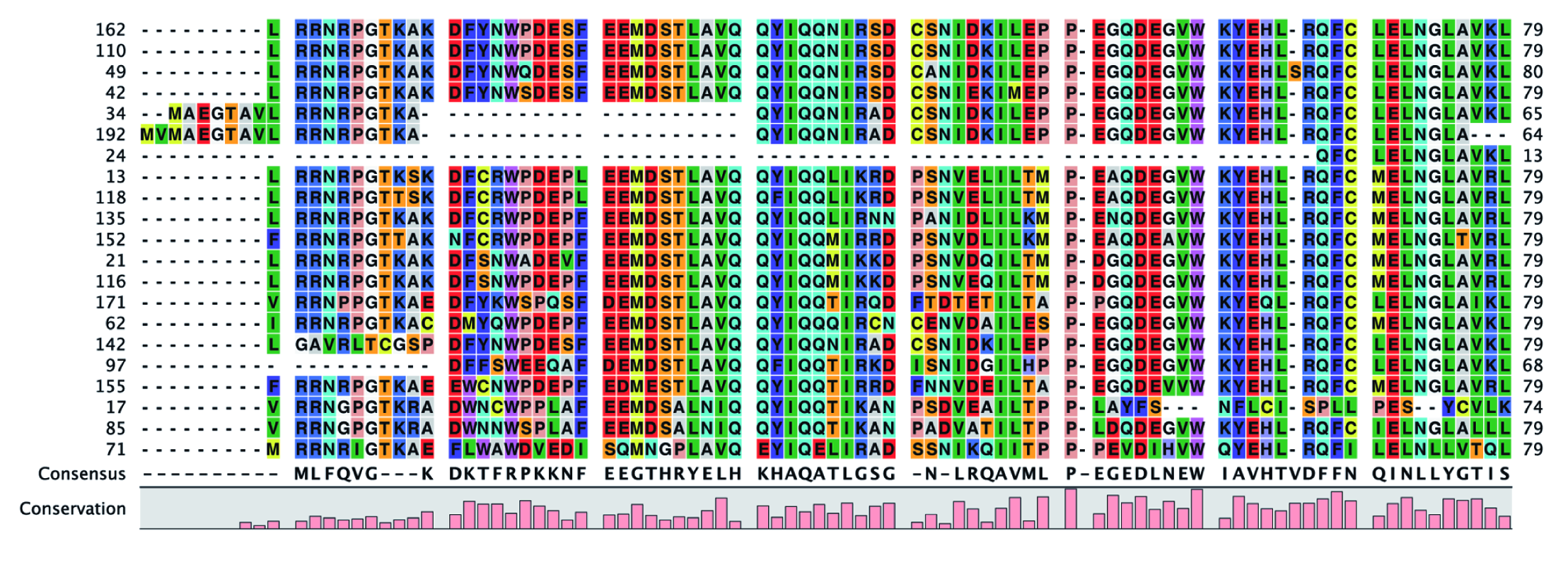

Supplement: Figure 1S. — Multiple alignment of the 192 Mob-domain containing proteins. The label number refers the fourth column in Table 1S of the supplementary materials and corresponds to the gene code. [file EBO-03-121-g00S3.tif]

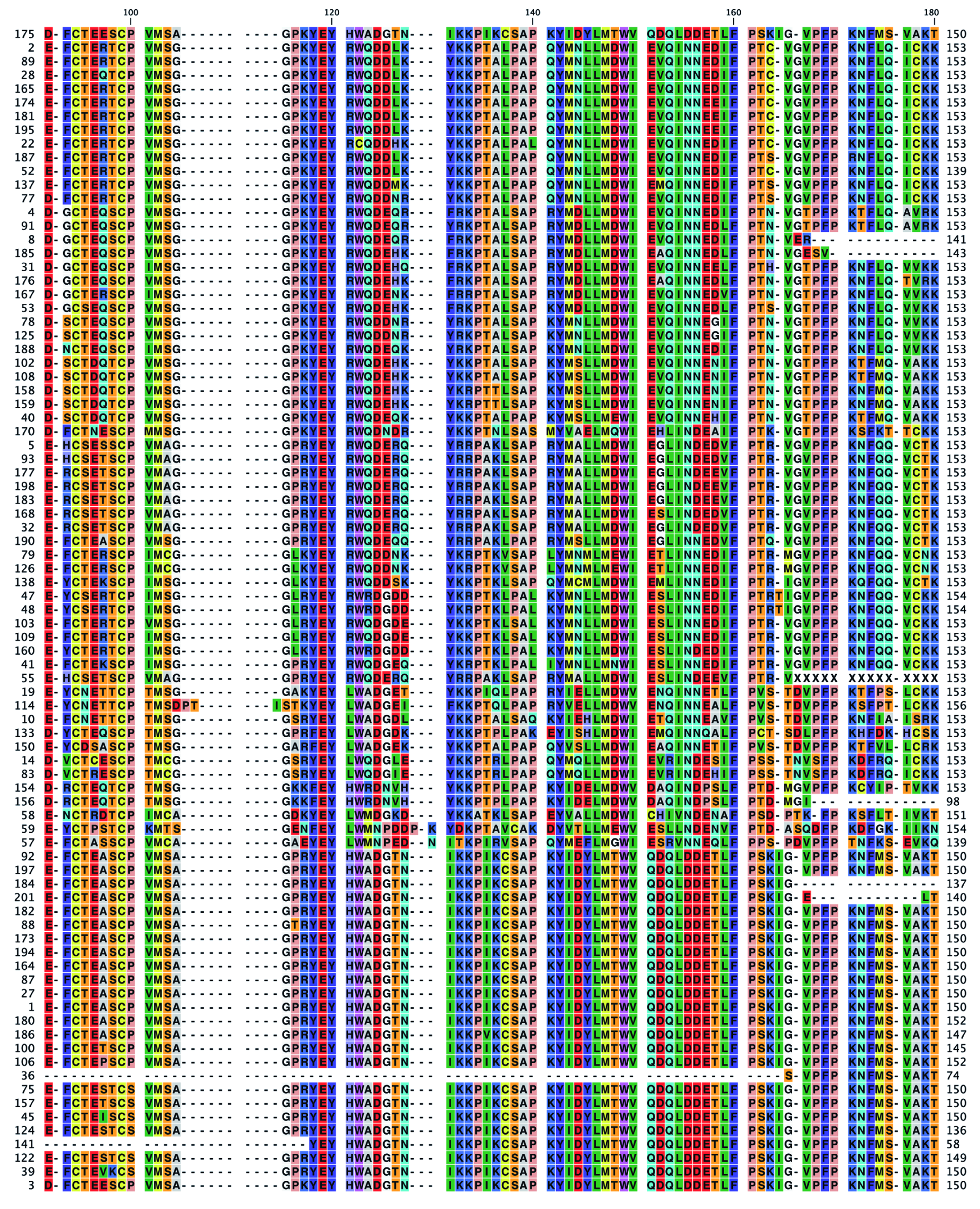

Supplement: Figure 1S. — Multiple alignment of the 192 Mob-domain containing proteins. The label number refers the fourth column in Table 1S of the supplementary materials and corresponds to the gene code. [file EBO-03-121-g00S4.tif]

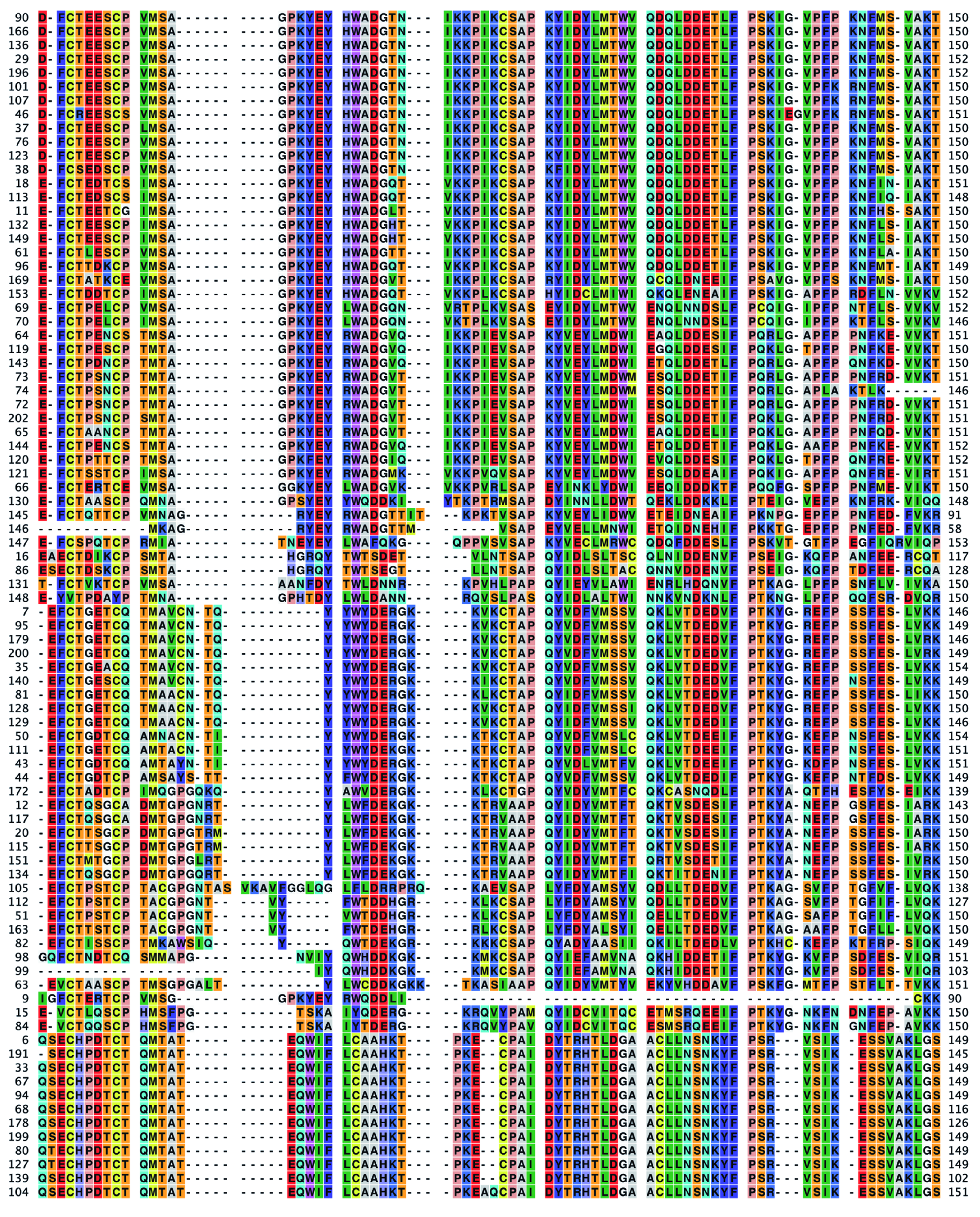

Supplement: Figure 1S. — Multiple alignment of the 192 Mob-domain containing proteins. The label number refers the fourth column in Table 1S of the supplementary materials and corresponds to the gene code. [file EBO-03-121-g00S5.tif]

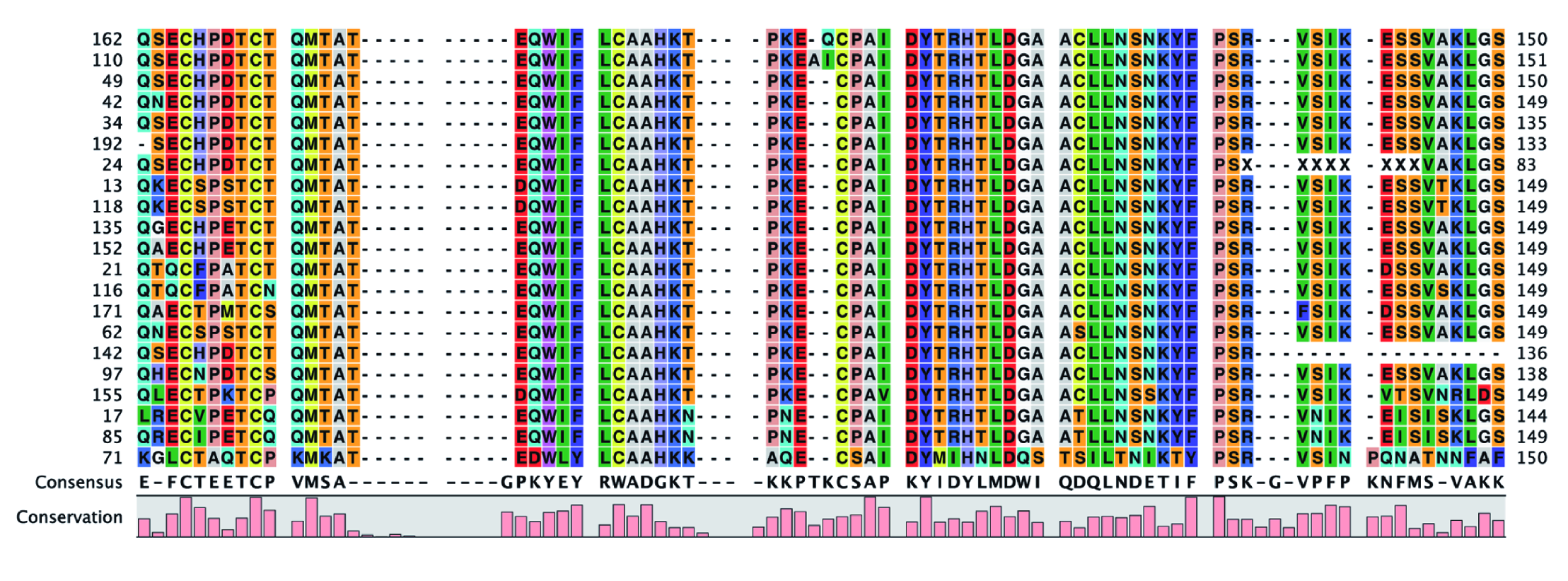

Supplement: Figure 1S. — Multiple alignment of the 192 Mob-domain containing proteins. The label number refers the fourth column in Table 1S of the supplementary materials and corresponds to the gene code. [file EBO-03-121-g00S6.tif]

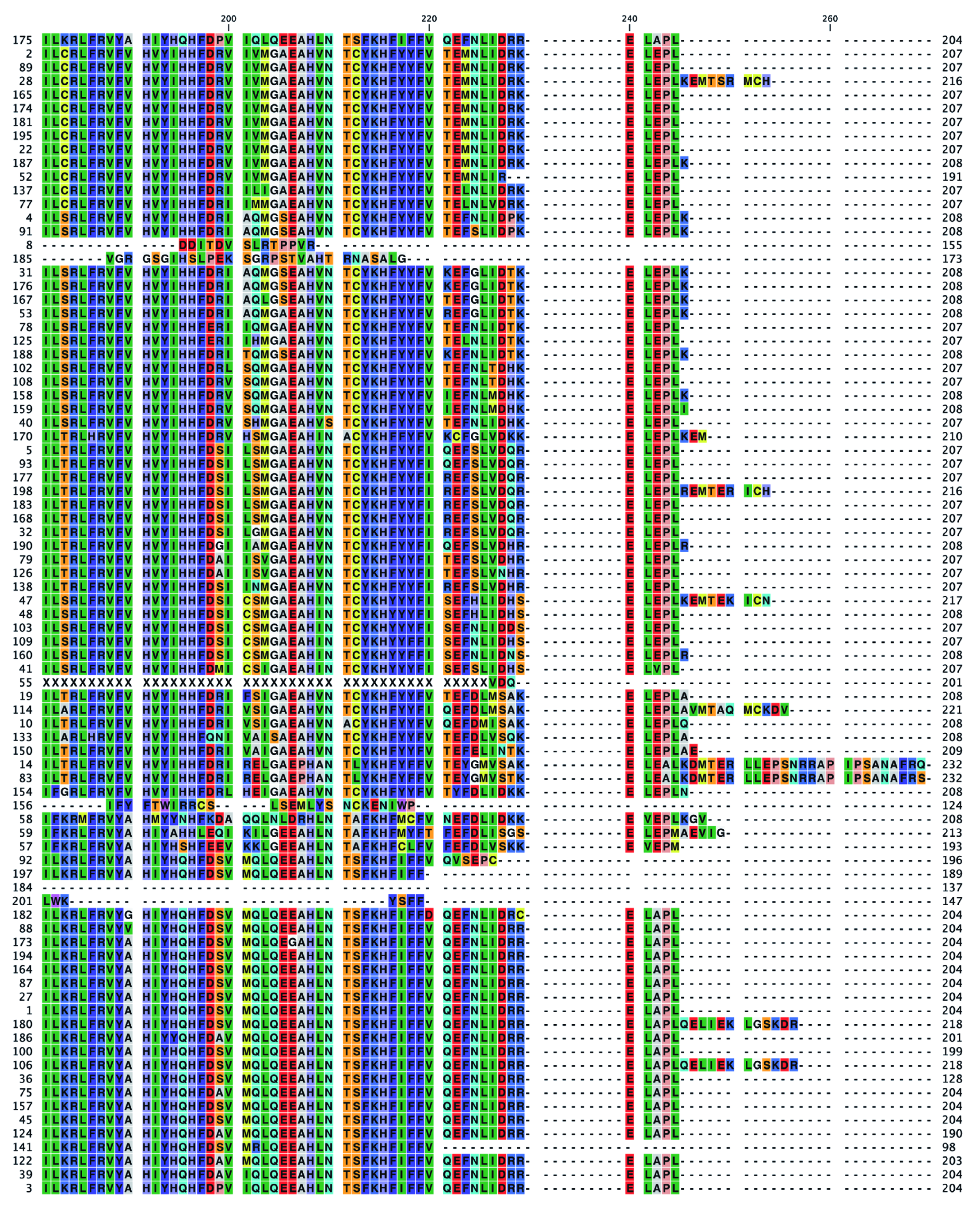

Supplement: Figure 1S. — Multiple alignment of the 192 Mob-domain containing proteins. The label number refers the fourth column in Table 1S of the supplementary materials and corresponds to the gene code. [file EBO-03-121-g00S7.tif]

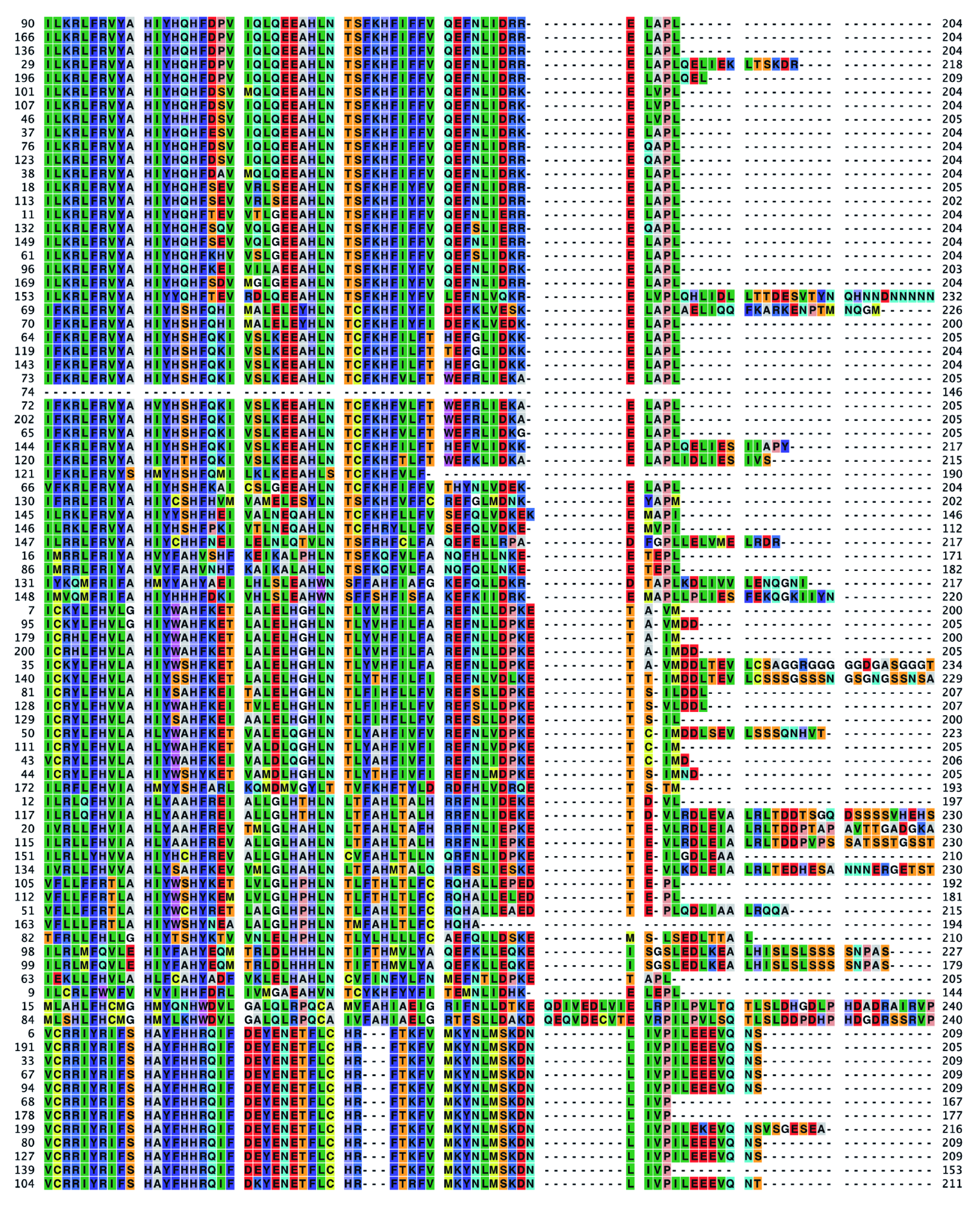

Supplement: Figure 1S. — Multiple alignment of the 192 Mob-domain containing proteins. The label number refers the fourth column in Table 1S of the supplementary materials and corresponds to the gene code. [file EBO-03-121-g00S8.tif]

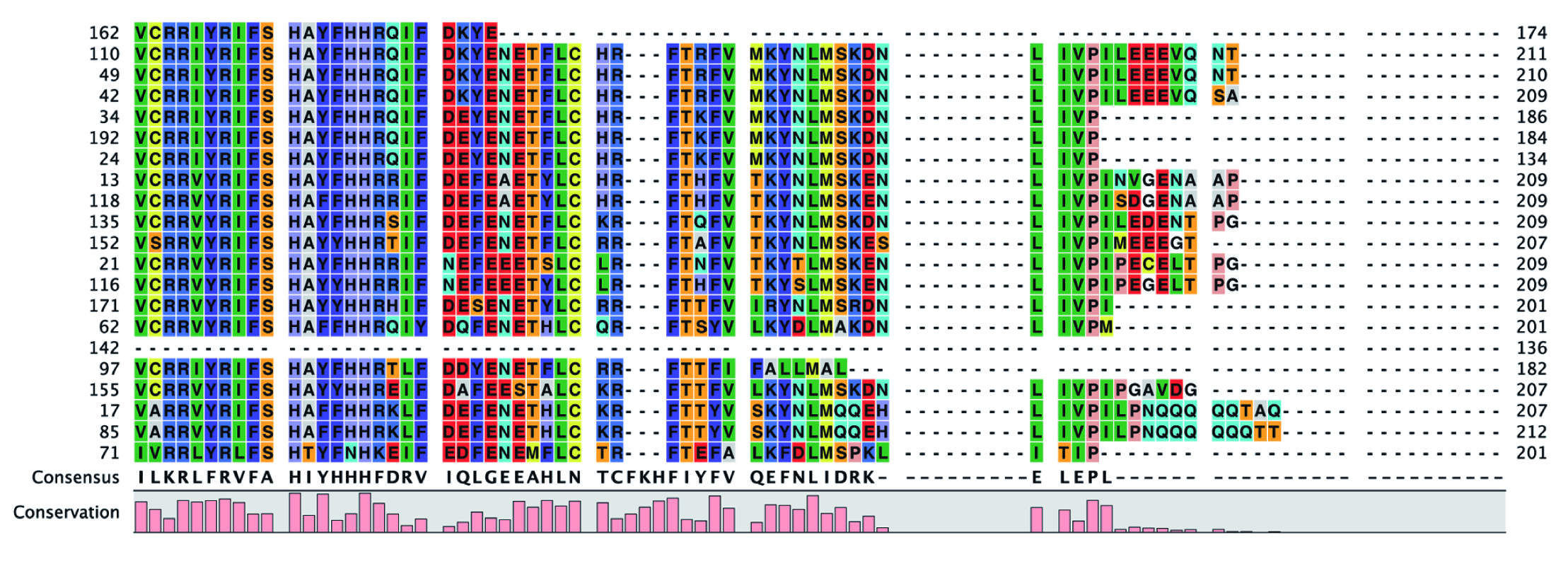

Supplement: Figure 1S. — Multiple alignment of the 192 Mob-domain containing proteins. The label number refers the fourth column in Table 1S of the supplementary materials and corresponds to the gene code. [file EBO-03-121-g00S9.tif]
